# Mortality Surveillance Methods to Identify and Characterize Deaths in Child Health and Mortality Prevention Surveillance Network Sites

**DOI:** 10.1093/cid/ciz599

**Published:** 2019-10-09

**Authors:** Navit T Salzberg, Kasthuri Sivalogan, Quique Bassat, Allan W Taylor, Sunday Adedini, Shams El Arifeen, Nega Assefa, Dianna M Blau, Richard Chawana, Carrie Jo Cain, Kevin P Cain, J Patrick Caneer, Mischka Garel, Emily S Gurley, Reinhard Kaiser, Karen L Kotloff, Inacio Mandomando, Timothy Morris, Peter Nyamthimba Onyango, Hossain M S Sazzad, J Anthony G Scott, Anna C Seale, Antonio Sitoe, Samba O Sow, Milagritos D Tapia, Ellen A Whitney, Mary Claire Worrell, Emily Zielinski-Gutierrez, Shabir A Madhi, Pratima L Raghunathan, Jeffrey P Koplan, Robert F Breiman, Child Health and Mortality Prevention Surveillance (CHAMPS) Methods Consortium, Janet Agaya, Victor Akelo, Beth A Tippett Barr, Sanwarul Bari, Farzana Islam, Afruna Rahman, Yadeta Dessie, Letta Gedefa, Erick Kaluma, Adama Mamby Keita, Uma U Onwuchekwa, Diakaridia Sidibe, Amara Jambai, Lola Madrid, Stefanie Wittmann, Ashka Mehta, Khátia Munguambe, Ariel Nhacolo, Pio Vitorino, Charfudin Sacoor, Jessica Preslar, Dickens Onyango, Jaume Ordi, Clara Menéndez Santos, Solomon Samura, Megan Turk, Rosauro Varo

**Affiliations:** 1 Emory Global Health Institute, Emory University, Atlanta, Georgia, USA; 2 ISGlobal, Hospital Clínic, Universitat de Barcelona, Spain; 3 Centro de Investigação em Saúde de Manhiça (CISM), Maputo, Mozambique; 4 Catalan Institution for Research and Advanced Studies (ICREA), Barcelona, Spain; 5 Pediatric Infectious Diseases Unit, Pediatrics Department, Hospital Sant Joan de Déu, Universitat de Barcelona, Spain; 6 Consorcio de Investigacion Biomedica en Red de Epidemiologia y Salud, Spain; 7 Center for Global Health, Centers for Disease Control and Prevention, Atlanta, Georgia, USA; 8 Medical Research Council, Respiratory and Meningeal Pathogens Research Unit, University of the Witwatersrand, Faculty of Health Sciences, Johannesburg, South Africa; 9 Department of Science and Technology/National Research Foundation: Vaccine Preventable Diseases, University of the Witwatersrand, Faculty of Health Sciences, Johannesburg, South Africa; 10 icddr,b, Dhaka, Bangladesh; 11 Maternal and Child Health Division, icddr,b, Dhaka, Bangladesh; 12 College of Health and Medical Sciences, Haramaya University, Harar, Ethiopia; 13 World Hope International, Makeni, Sierra Leone; 14 US Centers for Disease Control and Prevention–Kenya, Nairobi, Kenya; 15 Public Health Informatics Institute, The Task Force for Global Health, Atlanta, Georgia, USA; 16 Department of Epidemiology, Johns Hopkins Bloomberg School of Public Health, Baltimore, Maryland, USA; 17 US Centers for Disease Control and Prevention–Sierra Leone, Freetown, Sierra Leone; 18 Department of Pediatrics, Center for Vaccine Development and Global Health, University of Maryland School of Medicine, Baltimore, Maryland, USA; 19 Instituto Nacional de Saude, Ministerio de Saude, Maputo, Mozambique; 20 Kenya Medical Research Institute, Kisumu, Kenya; 21 University of New South Wales, Sydney, Australia; 22 PEI, Infectious Disease Division, icddr,b, Dhaka, Bangladesh; 23 Department of Infectious Disease Epidemiology, London School of Hygiene and Tropical Medicine, London, United Kingdom; 24 KEMRI-Wellcome Trust Research Programme, Kilifi, Kenya; 25 Centre pour le Développement des Vaccins (CVD-Mali), Ministère de la Santé, Bamako, Mali; 26 International Association of National Public Health Institutes, US Office at Emory Global Health Institute, Emory University, Atlanta, Georgia, USA

**Keywords:** CHAMPS, child mortality, global health, surveillance

## Abstract

Despite reductions over the past 2 decades, childhood mortality remains high in low- and middle-income countries in sub-Saharan Africa and South Asia. In these settings, children often die at home, without contact with the health system, and are neither accounted for, nor attributed with a cause of death. In addition, when cause of death determinations occur, they often use nonspecific methods. Consequently, findings from models currently utilized to build national and global estimates of causes of death are associated with substantial uncertainty. Higher-quality data would enable stakeholders to effectively target interventions for the leading causes of childhood mortality, a critical component to achieving the Sustainable Development Goals by eliminating preventable perinatal and childhood deaths. The Child Health and Mortality Prevention Surveillance (CHAMPS) Network tracks the causes of under-5 mortality and stillbirths at sites in sub-Saharan Africa and South Asia through comprehensive mortality surveillance, utilizing minimally invasive tissue sampling (MITS), postmortem laboratory and pathology testing, verbal autopsy, and clinical and demographic data. CHAMPS sites have established facility- and community-based mortality notification systems, which aim to report potentially eligible deaths, defined as under-5 deaths and stillbirths within a defined catchment area, within 24–36 hours so that MITS can be conducted quickly after death. Where MITS has been conducted, a final cause of death is determined by an expert review panel. Data on cause of death will be provided to local, national, and global stakeholders to inform strategies to reduce perinatal and childhood mortality in sub-Saharan Africa and South Asia.

## Teaser

This manuscript details methods for the establishment of mortality surveillance for timely detection of deaths in children <5 years of age and stillbirths in CHAMPS surveillance sites as well as data collection methods for notified and consented deaths.

Despite reductions over the past 2 decades, childhood mortality remains high in low- and middle-income countries (LMICs) in sub-Saharan Africa and South Asia. Children in these countries often die without a documented medical history, so the cause of death remains unknown [1]. Many countries with high child mortality have weak or nonexistent civil registration systems and death certificates are often not requested for stillbirths and children <5 years of age [2]. Those deaths that occur outside a health facility, especially stillbirths and deaths among neonates, who may be buried quickly, are likely to be missed from official statistics [3–5]. Even for children who die at a health facility, determining cause of death is difficult due to the scarcity of diagnostic tools and multiple coexisting illnesses, which often leads to an incorrect or nonspecific physician-ascribed cause of death [6, 7]. Research conducted in sub-Saharan Africa, the United States, and Europe indicates discordant diagnoses between autopsy findings and clinical diagnoses in as many as half of deaths [8, 9].

While complete diagnostic autopsies (CDA) are considered the most comprehensive method to determine cause of death [10], they are rarely conducted in LMICs due to cultural and religious acceptability issues as well as resource constraints [11–14]. A noninvasive substitute for CDA is the verbal autopsy (VA), a postmortem structured interview with individual(s) close to the deceased, which is recommended by the World Health Organization (WHO) [15–16]. However, VAs are hampered by a lack of objective diagnostic information and substantial recall bias and cannot reliably distinguish between many diseases with similar clinical presentations, something common to many childhood diseases [10, 17]. VA has particular difficulty in providing useful discriminatory information for deaths associated with congenital abnormalities [18], deaths in the perinatal period, and stillbirths [10]. VA algorithms for determining the cause of death are dependent on a priori knowledge of causes of death and therefore cannot investigate new causes of death. Finally, VAs are less and less useful for determining the residual causes of death in settings with decreasing mortality. The resulting data have limited value for decision making.

The Child Health and Mortality Prevention Surveillance (CHAMPS) Network was established to collect robust, standardized, longitudinal mortality data in a network of sites with the overarching objective of understanding and tracking preventable causes of childhood death in high mortality areas. CHAMPS aims to estimate overall and cause-specific mortality rates (stillbirth and under-5) in each site and extrapolate to regions with high child mortality beyond the catchment areas. The timely and accurate data generated by this initiative is intended to drive interventions to accelerate the reduction of childhood death and disability in low-resource settings. Sites aim to identify all under-5 deaths and stillbirths within the catchment area and conduct CHAMPS procedures, including postmortem minimally invasive tissue sampling (MITS) (also known as minimally invasive autopsy [MIA]), coupled with advanced laboratory techniques. The MITS procedure, a tissue and body fluid sampling method, was specifically developed to help increase acceptance of postmortem specimen testing in developing countries [19–22]. The procedure is more rapid, less invasive (and therefore, potentially more culturally acceptable), and less expensive than the CDA [14, 23–25], yet it generates equivalent information for many causes of death [19, 21, 24, 26–32]. For example, in a sample of stillbirths, the MITS procedure was shown to have substantial overall concordance (83%) with CDA cause of death determination. Among a sample of neonatal deaths, overall concordance was found to be 68%, with higher concordance shown in neonates with infectious diseases (85%) and preterm complications (60%) [29]. Additionally, overall concordance was found to be 72.7% in a study comparing MITS and CDA in fetuses [27]. As findings indicate that MITS should be done shortly after death to reduce the chance of tissue autolysis and postmortem overgrowth [33], sites have set up timely and comprehensive mortality detection systems to conduct most MITS within 24 hours of the death.

## DESIGN

**Figure 1.**
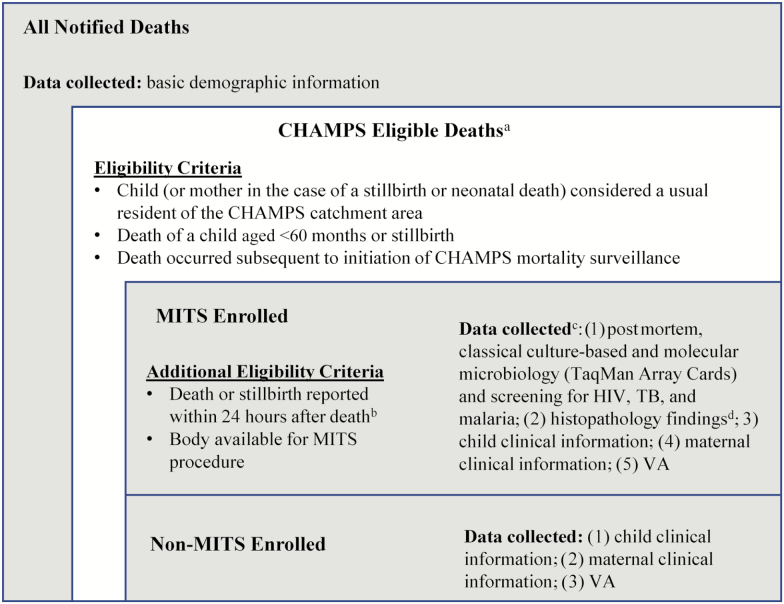
Inclusion and exclusion criteria for minimally invasive tissue sampling (MITS) and non-MITS enrollment. ^a^A small proportion of confirmed Child Health and Mortality Prevention Surveillance (CHAMPS) eligible deaths (ie, family was approached for eligibility screening and confirmed eligibility information) are not enrolled in CHAMPS due to parental nonconsent or loss to follow-up. ^b^The MITS timeframe may be extended up to 72 hours after death if body is refrigerated shortly after death. ^c^Circumstances may prevent the MITS from being conducted after MITS consent has been obtained. In these infrequent cases, data collection aligns with non-MITS procedures. ^d^Histology is conducted at the site and at the central pathology laboratory located at the US Centers for Disease Control and Prevention. Abbreviations: CHAMPS, Child Health and Mortality Prevention Surveillance; HIV, human immunodeficiency virus; MITS, minimally invasive tissue sampling; TB, tuberculosis; VA, verbal autopsy.

**Figure 2.**
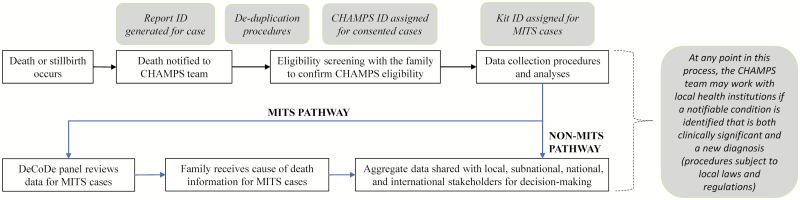
Overview of Child Health and Mortality Prevention Surveillance procedures. Abbreviations: CHAMPS, Child Health and Mortality Prevention Surveillance; DeCoDe, Determination of Cause of Death; ID, identifier; MITS, minimally invasive tissue sampling.

Site surveillance teams enumerate eligible under-5 deaths and stillbirths within the defined populations and approach families for consent for CHAMPS procedures (see Figure 1 for complete eligibility criteria and data collection categories). For eligible deaths notified within the MITS timeframe (within 24–36 hours of death or the body has been refrigerated), a MITS is conducted and additional data (clinical abstraction and VA) are collected. For these deaths (“MITS deaths”), a cause of death is determined by a Determination of Cause of Death (DeCoDe) expert panel that reviews all available data and seeks consensus to determine the most likely chain of causes or events leading to death [34]. For eligible deaths without a MITS performed (“non-MITS deaths”), a smaller subset of data is collected to allow adjustment and extrapolation of cause-specific mortality rates. A death may be enrolled in non-MITS if it is not notified within the MITS timeframe (and the body has not been refrigerated) or the parents refuse MITS. See Figure 2 for a high-level depiction of CHAMPS procedures.

### Study Population

**Figure 3.**
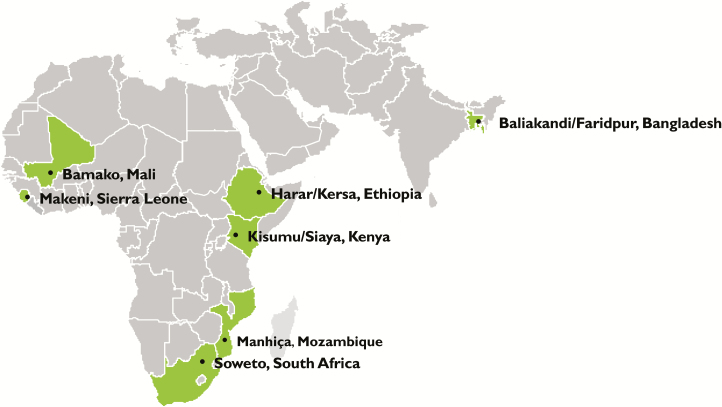
Map of Child Health and Mortality Prevention Surveillance sites. Abbreviation: CHAMPS, Child Health and Mortality Prevention Surveillance.

The CHAMPS Network consists of 7 sites in sub-Saharan Africa and South Asia, each with a geographically defined catchment area: Baliakandi and Faridpur, Bangladesh; Bamako (Djicoroni Para and Banconi), Mali; Kersa and Harar, Ethiopia; Makeni (Bombali Shebora and Bombali Siari Chiefdoms), Sierra Leone; Manhiça, Mozambique; Siaya (Karemo) and Kisumu (Manyatta), Kenya; and Soweto and Thembelihle, South Africa (Figure 3). Following an application process, these sites were selected based on a variety of factors, including history of conducting (or capacity to conduct) surveillance, under-5 mortality of >50 deaths per 1000 live births in children aged <5 years at the time of site selection (2015), and willingness of the local lead investigator to use a common, multisite protocol and to share data globally in real time; in addition, there was an intent to maximize geographic and ecologic diversity among the selected sites. A key consideration in site selection was the possibility for a strong relationship between the site and the local ministry of health and/or national public health institute, to ensure that data collected contribute to national public health policy and actions (Table 1). In addition, 2 sites (the sites in Sierra Leone and Ethiopia) were selected because of high child mortality and limited history of studies to understand disease burden.

**Table 1.** Selected Site Characteristics of Child Health and Mortality Prevention Surveillance Sites

Characteristic	Mozambique	South Africa	Mali	Kenya	Bangladesh	Ethiopia	Sierra Leone
Catchment area(s)	Manhiça	Soweto (selected 8 clusters), Thembelihle and surrounding informal settlements	Bamako (Djicoroni Para and Banconi)	Siaya County (Karemo) and Kisumu (Manyatta)	Baliakandi and Faridpur (selected 6 subclusters)	Kersa and Harar	Makeni (Bombali Shebora and Bombali Siari chiefdoms)
Setting	Rural	Urban	Urban	Rural (Siaya); urban (Kisumu)	Rural (Baliakandi); mixed (Faridpur)	Rural (Kersa); urban (Harar)	Urban and rural
HDSS establishment year	1996	2017 (will be fully established in 2019)	2006	2007 (Siaya); 2016 (Kisumu)	2017 (Baliakandi); no HDSS established (Faridpur)	2007 (Kersa); 2012 (Harar)	No HDSS established (early planning phase; currently using census data)
Total population under surveillance (2017)	186 000	123 225 (2018 HDSS)	87 126 (Djicoroni Para); 139 684 (Banconi) (2018 HDSS)	93 000 (Siaya); 72 000 (Kisumu)	216 362 (Baliakandi); ~2 000 000 (Faridpur 6 subclusters)	131 431 (Kersa); 50 000 (Harar)	161 383
Population density	81/km^2^	6400/km^2^	17 709/km^2^ (Djicoroni Para); 22 421/km^2^ (Banconi)	359/km^2^ (Siaya); est. 10 000/km^2^ (Kisumu)	894/km^2^ (Baliakandi); 1114/km^2^ (Faridpur 6 subclusters)	372/km^2^ (Kersa); 1244/km^2^ (Harar)	7521/km^2^; (Makeni City); 139.6/km^2^ (Bombali Shebora); no data for Bombali Siari alone
Under-5 population (2017)	26 425	12 962 (2018 HDSS)	13 631 (Djicoroni Para); 24 450 (Banconi) (2018 HDSS)	12 090 (Siaya); 11 700 (Kisumu)	20 180 (Baliakandi); ~180 000 (Faridpur 6 subclusters)	15 751 (Kersa); 4283 (Harar)	22 247
Government stakeholders	Instituto Nacional De Saúde (National Institute of Health)	Ministry appointed Committee for Morbidity and Mortality in Children, Gauteng Department of Health District Health Team; NICD	Ministry of Heath; INSTAT	Siaya and Kisumu county departments of health; Ministry of Health	Institute of Epidemiology, Disease Control, and Research; Ministry of Health and Social Welfare	Ethiopian Public Health Institute; Ministry of Heath	National Public Health Agency, currently under development; Sierra Leone Ministry of Health and Sanitation
Primary research partner(s)	Manhiça Health Research Centre; Barcelona Institute for Global Health (ISGlobal)	University of Witwatersrand; Medical Research Council: RMPRU	Center for Vaccine Development, University of Maryland, Baltimore	CDC Kenya; Kisumu County Department of Health; KEMRI	icddr,b; Bangabandhu Sheikh Mujib Medical University	Haramaya University; London School of Hygiene and Tropical Medicine	CDC–Sierra Leone; ICAP Columbia University; Focus 1000; World Hope International

Abbreviations: CDC, Centers for Disease Control and Prevention; est., estimated; HDSS, health and demographic surveillance system; INSTAT, Institut National de la Statistique (National Institute of Statistics); KEMRI, Kenya Medical Research Institute; NICD, National Institute for Communicable Diseases; RMPRU, Respiratory and Meningeal Pathogens Research Unit.

For 12–18 months before initiating CHAMPS activities, each site established a social-behavioral sciences (SBS) team to examine local social, cultural, and religious norms and to engage local communities to explain the project aims and methods and seek their support. Site SBS teams conduct focus groups, assist with rumor identification and mitigation, support families, and engage the community through a variety of activities [35, 36].

To monitor demographic trends and calculate mortality rates, most sites conduct surveillance within a health and demographic surveillance system (HDSS). An HDSS estimates the size and structure of a population by recording all births, deaths, and in- and out-migration episodes within a defined area. This is usually achieved through an initial census and intermittent reenumeration rounds 1–4 times per year. This information on key population indicators and characteristics, including fertility, mortality, and migration rates, also provides the total death count in the population to determine the level of ascertainment of deaths within the catchment area and explore biases in ascertainment. HDSS platforms vary in maturity throughout the network (Table 1) [37]. Several sites began surveillance with limited or no HDSS infrastructure, but these systems are being developed as part of the CHAMPS surveillance model. For sites with an existing HDSS, surveillance systems are being reviewed and strengthened (when necessary) to provide reliable denominators for rate calculations.

All sites have received approval from appropriate ethics review committees to conduct CHAMPS mortality surveillance. The CHAMPS Program Office (PO), based at Emory University, has received approval from the Emory University Institutional Review Board (IRB). In addition, since the initiation of CHAMPS, a team of ethicists from the Emory Center for Ethics conducts ongoing review of program practices and policies.

### Death Identification and Tracking

The CHAMPS identifier (ID) system accommodates surveillance at site and subsite levels of resolution. Reported deaths are issued a report ID to facilitate the tracking and linkage of limited data on reported deaths that may not ultimately get enrolled. Duplicate notifications are indicated as such in the database. Enrolled deaths and mothers with indicated consent for maternal abstraction are issued a unique CHAMPS ID; for a mother, the CHAMPS ID is associated with the pregnancy event related to the deceased child. Therefore, in the case of deceased twins, each twin is issued a unique CHAMPS ID and the mother is issued a single Pregnancy CHAMPS ID to associate the twins to a single pregnancy event. The mother’s information is also entered into a maternal registry that links all issued Pregnancy CHAMPS IDs. This event-based ID system allows the CHAMPS Network to link a single woman to multiple deceased children over time or link a child to different events throughout a lifetime, facilitating the addition of future research modules onto the CHAMPS platform. A unique MITS specimen collection kit ID is assigned to each MITS that is performed; each kit contains prelabeled collection containers with barcoded specimen ID labels along with labels for extra specimens. The specimen ID number is a combination of the specimen kit ID and a component ID that identifies the type of specimen collected. The linkage of CHAMPS ID to specimen kit ID enables the linking of any laboratory result to a specific MITS death.

### Mortality Surveillance and Death Notification

Each site has developed mortality surveillance and death notification procedures tailored to the needs of their catchment area and informed by SBS research, considering site capacity, cultural and religious norms and concerns, and geography (Tables 1–3) [35, 36]. See Table 4 for examples of how 2 sites are using a call center to receive and process death notifications. While some level of procedural variation prior to enrollment is supported, the PO has developed network-wide data quality and monitoring procedures to safeguard essential data elements collected upon notification and eligibility screening that may influence cause of death determination or are used in aggregate analyses (ie, basic demographics and details around the death). This approach allows sites to conduct surveillance in a contextually appropriate manner while also promoting network-wide standardization of key elements. While some sites have implemented both community and health facility–based notification systems, others have started notification activities first in a facility setting before implementing notification in the community (Table 3).

**Table 2.** Selected Health Characteristics of Child Health and Mortality Prevention Surveillance Sites

Characteristic	Mozambique	South Africa	Mali	Kenya	Bangladesh	Ethiopia	Sierra Leone
Mortality data at the time of site selection^a^							
U5MR (per 1000 live births)	71	56	123	76.6 (Siaya) and 79 (Kisumu)	50 (est.) (Baliakandi); unknown (Faridpur)	81.9 (Kersa); 22 (Harar)	156
IMR (per 1000 live births)	40.6	40	78	54	41 (Baliakandi); unknown (Faridpur)	45 (Kersa); 11 (Harar)	92
NMR (per 1000 live births)	15.6	21	40	39	30 (Baliakandi); unknown (Faridpur)	26.2 (Kersa); 4.1 (Harar)	39
SBR (per 1000 third-trimester pregnancies)	Unknown	23	28	Unknown	22 (Baliakandi); unknown (Faridpur)	15.7 (Kersa); 4 (Harar)	24.8 per 1000 live births^b^
MMR (per 100 000 live births)	208	310	550	495	Unknown	365	1360^c^; 695.7^d^
HIV							
Country HIV prevalence, age 15–49, % (year)	13.2 (2015)	18.9 (2017)	1.2 (2017)^e^	21 (Siaya, 2018); 16.3 (Kisumu, 2018)	<1 (2011)	1.1 (2016)	1.7 (2018)
Site HIV prevalence, age 15–49, % (year)	39.7 (2012)	10 (est.)	Unknown	Unknown	Unknown	Unknown	Unknown
HIV prevalence in women of reproductive age, % (year)	30	29	1.6 (2017)^e^	22.4 (Siaya, 2018); 17.4 (Kisumu, 2018)^f^	Unknown	Unknown	Unknown
Vertical mother-to-child transmission rate, %	5	1	Unknown^g^	Unknown	Unknown	Unknown	12.7
Malaria							
Endemic malaria	Yes	No	Yes	Yes	No	No	Yes

Abbreviations: est., estimated; HIV, human immunodeficiency virus; IMR, infant mortality rate; MMR, maternal mortality rate; NMR, neonatal mortality rate; SBR, stillbirth rate per 1000 births, unless otherwise indicated; U5MR, under-5 mortality rate.

^a^All data are from the point of Child Health and Mortality Prevention Surveillance (CHAMPS) site selection (as reported by the site) in 2015 unless otherwise indicated.

^b^GBD 2016 Mortality Collaborators. Global, regional, and national under-5 mortality, adult mortality, age-specific mortality, and life expectancy, 1970–2016: a systematic analysis for the Global Burden of Disease Study 2016. Lancet 2017; 390:1084–150.

^c^World Health Organization. Trends in maternal mortality: 1990 to 2015. Available at: *https://www.who.int/reproductivehealth/publications/.../maternal-mortality-2015/en/.*

^d^Global Burden of Disease Study. Global, regional, and national levels of maternal mortality, 1990–2015: a systematic analysis for the Global Burden of Disease Study 2015. Lancet 2016; 388:1775–812.

^e^Joint United Nations Programme on HIV/AIDS (UNAIDS). 2017 country factsheets, Mali. Available at: http://www.unaids.org/en/regionscountries/countries/mali.

^f^Rate from Jaramogi Oginga Odinga Teaching and Referral Hospital in Kisumu, Kenya.

^g^Based on UNAIDS 2017 country factsheet for Mali, 31% of pregnant women living with HIV received antiretroviral therapy for the prevention of vertical transmission.

**Table 3.** Selected Mortality Surveillance Characteristics of Child Health and Mortality Prevention Surveillance Sites

Characteristics	Mozambique	South Africa	Mali	Kenya	Bangladesh	Ethiopia	Sierra Leone
Mortality surveillance start date^a^	5 Dec 2016	22 Dec 2016 (Soweto); 1 Mar 2018 (Thembelihle)	1 Mar 2017	13 Sep 2017 (Siaya) and 24 May 2017 (Kisumu)	20 Sep 2017 (Baliakandi); 1 Oct 2018 (Faridpur)	4 Feb 2019	9 Oct 2017
MITS start date^b^	9 Dec 2016	22 Dec 2016	8 Aug 2017^c^	13 Sep 2017 (Siaya) and 24 May 2017 (Kisumu)	3 Oct 2017 (Baliakandi); 10 Oct 2018 (Faridpur)	4 Feb 2019	25 Feb 2019^c^
Phased death notifications^d^	No	Yes	No	No	No	No	No
Phased MITS enrollment^e^	Yes	Yes	No	No	Yes	Yes	Yes
Surveillance setting (as of June 2019)	Facility and community	Facility and community	Facility and community	Facility and community (Kisumu); community (Siaya)	Facility (Faridpur and Baliakandi)	Facility and community	Facility (MITS and non-MITS) and community (non-MITS only)
Surveillance staff	Health facility staff, CHWs, key community leaders	Health facility staff, community undertakers	Health facility staff, religious leaders, cemetery guards, CHWs (including relais and HDSS field workers), midwives	Health facility staff, mortuary staff, HDSS community members, religious leaders, CHWs, TBAs, village chiefs	Physician call center, health facility staff, religious leaders, community volunteers	Health facility staff, HDSS field workers, community reporters (CHWs, religious leaders, *kebele* leaders, traditional birth attendants)	117 call alert system, health facility staff, community reporters, mortuary staff
24-h mortality surveillance (notifications)	Yes	Yes	Yes	Yes	Yes	Yes	Yes
24-h consent and MITS	Yes	Yes	No	Yes	Yes	No	No
MITS facilities	Manhiça General Hospital; Hospital Rural de Xinavane	Chris Hani Baragwanath Academic Hospital	CVD-Mali Morgue	Jaramogi Oginga Odinga Teaching and Referral Hospital	Faridpur Medical College Hospital; Zahed Memorial Pediatrics Hospital; Baliakandi Upazila Health Complex	Hiwot Fana Hospital; Kersa Health Centre	Makeni Regional Hospital
Location of MITS procedure	Morgue	Morgue	Morgue	Morgue	MITS room in each hospital	Morgue and MITS room	Morgue
Family member allowed to view procedure	No	Yes	Yes	Yes	Yes	Yes	Yes
Complimentary studies	COMSA-Mozambique; CadMIA-Plus (Maputo)	…	…	Kenya Mortality Study	…	…	Social Autopsy Pilot; COMSA-Sierra Leone

Abbreviations: CaDMIA, Cause of Death using Minimally Invasive Autopsies; CHW, community health worker; COMSA, Countrywide Mortality Surveillance for Action; HDSS, health and demographic surveillance system; CVD, Center for Vaccine Development; MITS, minimally invasive tissue sampling; TBA, traditional birth attendant.

^a^The mortality surveillance start date is the date a site began collecting death notifications for Child Health and Mortality Prevention Surveillance network purposes.

^b^The MITS start date is the date a site began conducting MITS.

^c^Site began conducting non-MITS first before conducting MITS.

^d^Phased death notifications indicates that a site began mortality surveillance in a facility setting prior to conducting mortality surveillance in a community setting.

^e^Phased MITS enrollment indicates that a site began conducting MITS initially on facility-based deaths prior to conducting MITS on deaths that occur in the community.

**Table 4.** Call-Center Notification Systems

Faridpur/Baliakandi, Bangladesh	Bombali Shebora/Bombali Siari, Sierra Leone
The Bangladesh CHAMPS site developed a toll-free physician call center, partnering with MIAKI Media Ltd, to increase death reporting and also provide a service to the community. Through widely distributed health information materials, community members are instructed to call the center if they experience complications with pregnancy, child illness, or a medical emergency or would like newborn health advice. In case of illness, physicians discuss symptoms with callers to ascertain if there are any danger signs that require immediate referral to 1 of 6 designated health facilities. Community members are also instructed to call the center for reporting of births and child deaths. If a child mortality or stillbirth event is reported, relevant information is documented and communicated to the CHAMPS team. In addition to providing a platform for community death reporting, this system also enhances community access to physician consultation and improves the community’s abilities to make informed healthcare-seeking decisions.	The Sierra Leone CHAMPS site utilizes an existing toll-free call center to increase community-wide death reporting. The national emergency call system (known by its phone number, 117) was established in 2012 as part of a support system to improve maternal and child health and scaled up during the 2014–2016 Ebola outbreak for reporting all deaths and suspected cases of Ebola. Health providers and community members are instructed to call 117 to report all deaths. While overall reporting of deaths to 117 dropped sharply following the end of the Ebola epidemic, CHAMPS facility and community reporters have been trained to report child deaths and stillbirths to the call center, thereby increasing the system’s utilization and strengthening its utility. Additionally, the CHAMPS sociol-behavioral science team has conducted extensive community engagement to encourage widespread community use of the call center, addressing stigmatization that exists around the call center as a result of the Ebola epidemic. As a result of such activity, the CHAMPS catchment area Bombali District has the highest numbers of notified deaths to 117 in the country. When a child mortality or stillbirth event from within the CHAMPS catchment area is reported, relevant information is documented and communicated to the CHAMPS team. As the current civil registration and vital statistics data collection is paper-based, the 117 system provides an opportunity for creating an electronic reporting of deaths for civil registration and mortality surveillance programs.

Abbreviations: CHAMPS, Child Health and Mortality Prevention Surveillance.

To identify deaths occurring outside of health facilities, sites use a variety of community notification channels to enhance ongoing HDSS rounds (Table 3). Community reporters may be provided a cell phone and/or airtime to enable them to notify study staff of potentially eligible deaths in real time via short messaging service (text messaging) or phone calls. When applicable, efforts are made to ensure that community-based reporting is integrated into the site’s existing vital registration system, for example, by referring all deaths identified in the community to a health center to obtain a death certificate prior to enrollment. CHAMPS actively monitors timeliness, completeness, and representativeness of death notifications from facilities and the community through standardized metrics and data dashboards with the intent of improving representativeness over time. For instance, through monitoring death notifications stratified by age group, MITS eligibility, and location of death, the Mali site identified that a majority of notified stillbirths were already buried at the time of notification, making them ineligible for MITS. A few of these stillbirths were delivered in a health facility with a round-the-clock surveillance team. Based on this information, staff met with midwives and pediatricians to reintroduce CHAMPS, emphasize the importance of complete and timely reporting of stillbirths and under-5 deaths, and reinforce that midwives and other health facility personnel would not be blamed for stillbirths. The site experienced a subsequent increase in timely reporting of stillbirths in the following months.

#### Mortality Surveillance Data Collection

Upon the notification of a death, surveillance teams collect basic information to identify duplicate death notifications and to assess eligibility (ie, dates of birth and death, time of death, residency within the catchment area). All information is confirmed with the family prior to enrollment.

### Enrollment Procedures

For deaths identified within the MITS eligibility timeframe (within 24-36 hours of death or the body has been refrigerated), a consenting team is dispatched to the family as soon as possible to confirm eligibility. The consenting team is comprised of a health worker and staff trained to provide appropriate support and/or counseling to parents or family members. Before data are collected, a family member or guardian must give consent to participate in data collection. Consent procedures have been adapted at each site based on local SBS data [36].

## ENROLLED DEATH DATA COLLECTION

### MITS Procedure

After enrollment, the body is transported to a designated MITS procedure room within a local facility, and a series of tissue (ie, brain, both lungs, liver, bone marrow, heart) and nontissue specimens (ie, blood, cerebrospinal fluid, stool via rectal swabs, and respiratory secretions via nasopharyngeal/oropharyngeal swabs) are collected [38]. For stillbirths and neonatal deaths, specimens from the placenta, membranes, and umbilical cord are also collected, when available. If deemed culturally appropriate, a family member or designee may attend the MITS procedure if desired (Table 3). Following the completion of the procedure, which typically takes 1 hour to complete, the body is transported back to a nearby location as requested by the family.

### Child and Maternal Clinical Data Abstraction

Available clinical information pertaining to the deceased child as well as relevant maternal health information for a subset of deaths is abstracted for all enrolled deaths (MITS and non-MITS) (Table 5). The completeness of the data available varies greatly by site. Clinical records may be obtained from all levels of health facilities consulted during the course of the terminal illness/event or antenatal care clinics, or may be held by family members.

**Table 5.** Child and Maternal Health Information Abstracted for Minimally Invasive Tissue Sampling (MITS) and Non-MITS Cases

Form	Data Category
Child abstraction	Basic case information
	Recent hospital encounters and hospitalization leading to death
	Physical examination
	Past medical history
	Birth history
	Immunization records
	Growth chart
	Child and maternal HIV and TB information
	Diagnostic information
	Clinical summary^a^
Maternal abstraction^b^	Maternal demographic information
	Antenatal clinic history
	Pregnancy, labor, and delivery
	Placenta and cord description
	Maternal laboratory testing and treatments
	Maternal medications
	Maternal transfusions
	Information of previous pregnancies and pregnancy outcomes
	Perinatal outcome and basic characteristics of the enrolled deceased child
	Maternal death information (if applicable)

Abbreviations: HIV, human immunodeficiency virus; TB, tuberculosis.

^a^Clinical summary may be transcribed directly from patient records (if available) or may be composed by clinically experienced abstractors based upon available clinical data.

^b^Maternal records related to the deceased enrolled child are abstracted.

Clinical information on the delivery and maternal health is abstracted for all stillbirths or deaths among infants aged <1 year. It is also abstracted for deaths among children aged 1 year or older if the mother experienced complications or health problems during the pregnancy, labor, birth, or immediate postpartum period of the child in question.

### Verbal Autopsy

For all enrolled deaths, consent is requested to conduct a VA interview with a parent, other family member, or caregiver close to the child. This standardized interview, ideally conducted 2–4 weeks after the death, is intended to elucidate symptoms and signs related to the most common causes of death. Trained interviewers administer the WHO 2016 VA questionnaire, adapted to capture CHAMPS and HDSS identifiers and modified to include content enhancements, skip logic, and unit of measurement corrections (see Supplementary Materials). Additionally, CHAMPS developed a software application to receive and validate multiple VA versions in the field; some sites initially used the WHO 2012 VA instrument [39], and subsequently transitioned to the WHO 2016 VA.

Verbal autopsy data are analyzed by automated diagnostic algorithms, such as openVA [40], for statistical assignment of probable causes of death and for assessing ways to improve VA procedures and interpretations when compared to DeCoDe-generated cause of death determinations for MITS deaths. The automated VA conclusions are not provided to the DeCoDe panel, which instead receives the individual VA responses.

## CAUSE OF DEATH DETERMINATION PROCESS

The DeCoDe panel comprises at least 1 of each of the following: clinician (eg, pediatricians, neonatologists, and obstetricians), pathologist, epidemiologist, and microbiologist. Panelists receive all available information from linked maternal data, child clinical data, individual demographic data, VA, microbiology, molecular testing, clinical diagnostics (human immunodeficiency virus [HIV], tuberculosis, malaria), photographs of the deceased from the MITS procedure, and histopathology findings in the form of a DeCoDe packet and assign underlying, antemortem, and immediate (and other contributing) causes of death [34]. A unique version identifier and date/time stamp is created each time a DeCoDe packet is created, enabling regeneration of the packet if new information is added or a new panel reviews and provides another set of cause of death results.

## COMMUNICATING RESULTS

For deaths with a MITS performed, sites communicate this cause(s) of death to family members. The objective is to deliver results within 4 months of the death; however, a variety of challenges may delay this process. For instance, urban communities are highly transient and therefore, families are difficult to trace. Additionally, some DeCoDe panel assessments are delayed by the complexity of the necessary analyses.

Site staff communicate DeCoDe results to parent/guardians using understandable language and visual aids when appropriate. Findings may include negative test results as appropriate (such as indicating that the child did not have malaria or HIV); in some cases, the cause of death remains undetermined. If measures are identified that could have potentially prevented the child’s death (eg, timing of care seeking, danger signs), these are discussed with the family. Several sites conduct either follow-up interviews with participating families or observe the delivery of cause of death information to improve the process of delivering results to families. Diagnoses (such as tuberculosis or HIV) with clear public health implications or statutory reporting requirements are provided to local public health staff for follow-up. In some cases, it is necessary to communicate with families and other relevant parties before the DeCoDe results are available, based on a clinically relevant laboratory result (ie, positive for syphilis, HIV, tuberculosis, meningococcus, or cholera). In these cases, the site notifies the local public health authority, based on local reporting requirements, as well as notifies the family and treating clinician/facility. Additionally, local law enforcement may be notified in cases where child maltreatment is identified.

Findings are also shared with communities, local government, public health authorities, and facilities to guide public health action to prevent future child deaths and illness (Table 6). To facilitate this process, the International Association of National Public Health Institutes works with national and subnational public health authorities associated with the sites to ensure they have the skills and tools necessary to interpret and use CHAMPS data and integrate it with other sources of child mortality data with the objective of informing public health guidelines, resource allocation, policies, programs, communication strategies, and interventions.

**Table 6.** Child Health and Mortality Prevention Surveillance as a Mechanism for Data to Action

Soweto, South Africa	Manyatta, Kisumu County, Kenya
In South Africa, CHAMPS mortality surveillance was first launched in Chris Hani Baragwanath Hospital, a central hospital in Soweto. Based on initial DeCoDe data from these hospital-based cases, hospital-acquired infections were identified as a leading cause of neonatal mortality in that site. As a result, the South Africa CHAMPS team, in partnership with the United States CDC, led a critical appraisal of current facility infection prevention and control practices and subsequently, received a grant from the Bill & Melinda Gates Foundation to develop a package of interventions to mitigate against the risk of hospital infections. Additionally, based on the initial round of DeCoDe data, the site identified that approximately 20% of stillbirths in the South Africa site may be due to invasive bacterial infection of the fetus. This has led to increased sensitivity of facility-based obstetricians to the value in stillbirth investigation and has invigorated a research agenda aimed at addressing the burden of stillbirths in South Africa, a significant, yet largely ignored population. Through the review of data and evaluation of potential causes and mitigation factors, the CHAMPS South Africa site is ensuring that CHAMPS data will not only contribute toward a better understanding of child mortality but will also actively improve the overall health of its population.	The Kenya CHAMPS site, in collaboration with the Kisumu County MOH, recently established an HDSS system in Manyatta aimed at strengthening existing MOH surveillance by systematizing and improving documentation. Establishment of the HDSS included mapping of households and enumerating residents through a baseline survey that established a unique identifier for each individual. According to the MOH, the area covered by each CHV should include between 100 and 120 households. Mapping demonstrated that areas allocated to CHVs had significantly more households than previously thought; some had from 250 to 650 households and one CHV village had >800 households. The implications of the underestimation mean that CHVs only visited the first 100–120 households in their coverage area and stopped there, returning to the same households on subsequent visits. This impacts intervention planning since only a fraction of the population would potentially be reached. As an example, bed nets were distributed to households in Manyatta, yet many households reported not receiving one due to the underestimation of the need. Based on the information gathered through the HDSS mapping and enumeration, the coverage area allocated to each CHV was examined by Kisumu County MOH. The process of realigning the areas allocated to CHVs to conform to a maximum of 120 households and recruitment of additional CHVs is ongoing. The mapping and enumeration will allow CHAMPS to have an accurate denominator for calculating rates of under-5 mortality and also provide adequate coverage by CHVs for all the households.

Abbreviations: CDC, Centers for Disease Control and Prevention; CHAMPS, Child Health and Mortality Prevention Surveillance; CHV, community health volunteer; DeCoDe, Determination of Causes of Death; HDSS, health and demographic surveillance system; MOH, Ministry of Health.

## TRAINING

Site mortality surveillance staff are trained according to their responsibilities. The PO developed training modules that are adapted according to site-specific procedures and requirements. Training modules include death identification procedures, assignment of CHAMPS IDs, linkage and de-duplication of data, death notification, enrollment and consent, loss and grief training, compassionate interview skills, general biosafety procedures (including universal precautions and use of personal protective equipment), confidentiality and ethics, VA procedures, and abstraction procedures. Additionally, in collaboration with sites and the PO, ISGlobal and the Centers for Disease Control and Prevention conduct trainings for MITS specimen collection, histopathology, telepathology and TaqMan Array Card assays [38, 41]. Sites conduct training evaluations and retrain as necessary.

## STATISTICAL ANALYSIS AND DATA ACCESSIBILITY

Sites aim to enumerate and approach families of all eligible deaths for consent, whether MITS or non-MITS. The number of deaths with a MITS performed is determined by community acceptability, birth cohort and under-5 population, and number of deaths rather than theoretical considerations or power calculations. As a result of both the complexities involved with conducting community-based mortality surveillance and consent for MITS, the majority of enrolled deaths (particularly MITS deaths) during the initial phase of CHAMPS were identified through health facilities. Therefore, initial results may not be representative of the catchment population, as many deaths occur at home and may have different causes of death than facility-based deaths. As surveillance systems mature and a greater proportion of deaths occurring outside of facilities undergo MITS, the results will become more representative of the study populations.

CHAMPS will describe relative contributions to under-5 mortality of conditions and specific etiologies (eg, diarrheal disease, respiratory illness, febrile illness and associated pathogens, as well as noninfectious diseases) stratified by age group (eg, stillbirth and neonatal mortality) by site and across sites. Data collected will allow for the consideration of a variety of contributors to death, whenever available, including levels of illness severity, delays in accessing care, quality of care, general healthcare access, host factors (including malnutrition and inheritable diseases, like hemoglobinopathies), and comorbid conditions. As data accumulate, the network will extrapolate to estimate regional burden of specific causes of death. In addition, CHAMPS data are accessible to the scientific, clinical, and public health communities through its website (www.champshealth.org).

## DISCUSSION

CHAMPS is an ambitious, longitudinal, mortality surveillance project that will determine the causes of child mortality in 7 sites in sub-Saharan Africa and South Asia, regions with high child mortality and limited capacity to generate specific cause of death information. From program inception, sites have collaborated closely with local government and public health officials to ensure that data collected are available to influence recommendations, policies, and interventions (Table 6). Additionally, aggregate data are publicly accessible via the CHAMPS website, with more detailed data available upon request and IRB approval (as required). As mortality detection systems are strengthened and sites capture proportionately more of the deaths that occur within their catchment populations, the scope for bias will decrease and the results will be more representative. Additionally, the accumulation of deaths with a DeCoDe panel cause of death determination (projected 10 000 by 2025) will allow for a better understanding and contextualization of laboratory findings (eg, the role of coinfections in mortality, relative importance of certain pathogens in the mortality chain) and improved interpretation of VA results.

While CHAMPS has the potential to contribute significantly to the understanding of all-cause child mortality, common challenges arise from conducting multisite mortality surveillance. Although sites aim to enumerate all eligible under-5 deaths and stillbirths within their catchment area, many site HDSS systems do not yet have a precise way of identifying all eligible deaths in the catchment area and therefore, defining complete ascertainment is challenging. As sites continue to invest in the development and strengthening of their HDSS systems (when needed) and as enrolled deaths accumulate, reliability and confidence in site-level population denominators will increase and sites will have more accurate measurements of their success in complete mortality detection.

A variety of challenges limit the network’s current ability to extrapolate and generalize results. The representativeness of the data is limited without a full understanding of the deaths that may be missed by a surveillance system, particularly if those deaths are biased for particular factors (ie, access to healthcare, socioeconomic factors, demographic characteristics, criminal/legal potential that prohibits inclusion). Sites currently capture a larger proportion of deaths that occur within facilities than those that occur within the community. There is limited generalizability to areas outside of the catchment area, as the catchment area may not be representative of the rest of the host country or other similar areas. CHAMPS is developing methods based on Bayesian techniques to extrapolate from such potentially nonrepresentative samples, using data collected from the underlying population, such as age, sex, location, and season of death (to be described in a future publication).

Additionally, the MITS procedure has limited direct utility in determining cause of death from certain causes, including trauma, noninfectious causes of death such as congenital heart disease, occult congenital abnormalities, and genetic disorders not easily detectable by targeted specimen collection or photography. Localized organ lesions may not be appropriately sampled with fine needles through the “blind” biopsy methodology employed in the MITS procedure (ie, the needle did not enter the area of the infection). However, the MITS procedure has proven to be a widely acceptable method of postmortem examination in areas where full autopsies are not feasible and compared with CDA, the procedure has been shown to be highly accurate and sensitive [14, 23, 24, 29].

Routinely collecting specimens from bodies of dead children requires understanding of cultural, familial, and religious beliefs around death. Staff must be sensitive to the shock and grief of a family in response to the child’s death. In anticipation of these challenges, site SBS teams conduct activities to better understand the feasibility of CHAMPS procedures, beginning their work prior to launching mortality surveillance and continuing during program implementation [36]. Site community engagement teams work with local communities and stakeholders to address misconceptions about CHAMPS, and MITS in particular, in a timely manner, to increase and sustain the acceptability of MITS [35].

These challenges and limitations also provide opportunities for growth. SBS activities have been a key component to each site’s project implementation, with community engagement and formative research impacting how data are collected and how staff interact with community members. For instance, in Kenya, the SBS team identified that black cars, often used to transport the dead, were perceived by the community as an omen of death. This led to the site modifying their approach to body transportation and no longer using black cars to transport a body. In Bangladesh, consent procedures were adapted to engage the broader family network (rather than just the mother or father) based on SBS assessments that revealed that consent rates rose when the broader family could decide together whether to participate in CHAMPS. The incorporation of SBS findings into project implementation helps promotes community ownership over the CHAMPS program and ultimately can lead to greater participation and potential to use generated data for local and regional action. Many sites have formed community advisory boards that provide insight into how activities should align with community priorities. Local project champions help increase acceptance of the program as well as promote a better understanding of the program’s ultimate goals within their communities [35]. Additionally, the integration of family feedback and follow-up into the project will improve our understanding of the impact that data have on individual family members and surrounding communities. Finally, the wide network of sites has allowed for the ultimate improvement of cross-network tools (eg, 2016 WHO VA) and procedures.

Despite known and potential limitations and the expected challenges, by 20 June 2019, 1276 MITS were conducted from across the 7 CHAMPS sites and DeCoDe panels have met and completed cause of death determinations for 860 of these deaths [42]. Despite limited numbers, these determinations have already provided insights on contributions of key pathogens (eg, respiratory syncytial virus, malaria, *Streptococcus pneumoniae*, group B *Streptococcus*, malnutrition, preterm birth, and congenital abnormalities) within the causal chain of child mortality. As enrolled deaths accumulate and confidence in site-level population denominators increases through strengthening the HDSS [37], CHAMPS will provide specificity for how specific causes of death contribute to mortality burden. Data generated from CHAMPS will ultimately inform potential strategies to achieve the United Nations Sustainable Development Goal 3.2 to reduce the high stillbirth and under-5 child mortality seen in Africa and South Asia.

## Supplementary Data

Supplementary materials are available at *Clinical Infectious Diseases* online. Consisting of data provided by the authors to benefit the reader, the posted materials are not copyedited and are the sole responsibility of the authors, so questions or comments should be addressed to the corresponding author.

ciz599_suppl_Supplementary_Material

## Notes

***Child Health and Mortality Prevention Surveillance (CHAMPS) Methods Consortium.*** Janet Agaya (Kenya Medical Research Institute [KEMRI], Kisumu, Kenya); Victor Akelo and Beth A. Tippett Barr (US Centers for Disease Control and Prevention [CDC]–Kenya, Kisumu, Kenya); Sanwarul Bari (Maternal and Child Health Division, icddr,b, Dhaka, Bangladesh); Farzana Islam (PEI, Infectious Disease Division, icddr,b, Dhaka, Bangladesh); Afruna Rahman (PEI, Infectious Disease Division, icddr,b, Dhaka, Bangladesh); Yadeta Dessie and Letta Gedefa (College of Health and Medical Sciences, Haramaya University, Harar, Ethiopia); Erick Kaluma (Crown Agents, Makeni, Sierra Leone); Adama Mamby Keita, Uma U. Onwuchekwa , and Diakaridia Sidibe (Centre pour le Développement des Vaccins (CVD-Mali), Ministère de la Santé, Bamako, Mali); Amara Jambai (Ministry of Health and Sanitation, Sierra Leone); Lola Madrid and Stefanie Wittmann (Department of Infectious Disease Epidemiology, London School of Hygiene and Tropical Medicine, United Kingdom, and College of Health and Medical Sciences, Haramaya University, Harar, Ethiopia); Ashka Mehta (Department of Pediatrics, Center for Vaccine Development and Global Health, University of Maryland School of Medicine, Baltimore); Khátia Munguambe, Ariel Nhacolo, Pio Vitorino, and Charfudin Sacoor (Centro de Investigação em Saúde de Manhiça [CISM], Maputo, Mozambique); Khátia Munguambe (Eduardo Mondlane University, Faculty of Medicine, Community Health Department, Maputo, Mozambique); Jessica Preslar (Department of Epidemiology, Rollins School of Public Health, Emory University, Atlanta, Georgia); Dickens Onyango (Kisumu County Department of Health, Kisumu, Kenya); Jaume Ordi, and Clara Menéndez Santos (ISGlobal, Hospital Clínic, Universitat de Barcelona, Spain); Solomon Samura (World Hope International, Makeni, Sierra Leone); Megan Turk (Data Solutions, Library and Information Technology Services, Emory University, Atlanta, Georgia); and Rosauro Varo (ISGlobal, Hospital Clínic, Universitat de Barcelona, Spain, and CISM, Maputo, Mozambique).

***Acknowledgments.*** The authors thank the entire CHAMPS Network staff for their dedication and for contributing their insight and expertise to the project. In particular, the authors thank the following for their contributions to the project: the physician call-in center in Bangladesh; the CHAMPS project team, investigators and Scientific Advisory Committee in Ethiopia, who have all contributed to setting up this work; the large supporting team at the KEMRI–Centre for Global Health Research and the technical advisors of the CDC–Kenya; all of the CISM staff in Mozambique, particularly those supporting the CHAMPS program; the CDC–Sierra Leone for their support in setting up CHAMPS in Sierra Leone; Siobhan Johnstone for assisting in setting up the CHAMPS structure in South Africa and Nellie Myburgh for leading community engagement efforts in South Africa; and the extended CHAMPS team in Mali, including Aliou Toure, Professor Cheick Bougadari Traore, the minimally invasive tissue sampling and histopathology team, the microbiology and immunology departments, the demographic surveillance system (DSS) and sociobehavioral science teams, and the Determination of Cause of Death (DeCoDe) panelists. The authors thank the government of Mali, the Ministry of Health of Mali, Malian community leaders (religious, social, traditional), and Point Sud. The authors also thank Kevin Clarke from the US CDC, and Dave Ross and Vivian Singletary from the Task Force for Global Health. We want to extend our deepest appreciation to the communities of each CHAMPS site and the family members of enrolled deaths in particular who have supported CHAMPS work to define what is killing children. ISGlobal is a member of the Catalan Research Centres Institute (CERCA) Programme, Generalitat de Catalunya (http://cerca.cat/en/suma/). CISM receives support from the Government of Mozambique and the Spanish Agency for International Cooperation and Development. The Kersa HDSS has been receiving financial and technical support from the US CDC (grant number GH001039-01-05) and the Ethiopian Public Health Association.

***Financial support.*** This work was supported by the Bill & Melinda Gates Foundation (award number OPP1126780). J. A. G. S. is funded by a fellowship from the Wellcome Trust (award number 098532). CHAMPS is funded by the Bill & Melinda Gates Foundation, which has provided input into site selection decisions and methodology and scope of CHAMPS.

***Supplement sponsorship.*** This supplement is sponsored by the Emory Global Health Institute and the Bill & Melinda Gates Foundation.

***Potential conflicts of interest.*** S. A. M. has received grants from the Bill & Melinda Gates Foundation, Pfizer, Glaxo Smith Kline, Biovac, Novavax, and Sanofi Pasteur; and honoraria from The Bill & Melinda Gates Foundation. All authors report no potential conflicts of interest. All authors have submitted the ICMJE Form for Disclosure of Potential Conflicts of Interest. Conflicts that the editors consider relevant to the content of the manuscript have been disclosed.
