# Supplementary material for: Mortality Surveillance Methods to Identify and Characterize Deaths in Child Health and Mortality Prevention Surveillance Network Sites
Source: Clin Infect Dis. 2019 Oct 9;69(Suppl 4):S262–73. doi: 10.1093/cid/ciz599 (PMC6785672; doi:10.1093/cid/ciz599)
Supplement: ciz599_suppl_Supplementary_Material [file ciz599_suppl_supplementary_material.pdf]

CHAMPS Verbal Autopsy
Sample Questionnaire

Death of a child aged under four weeks

Powered by CHAMPS Engine

ID	Question	Answer(s)	Skip
alt_id_1	Do you have any alternate identifiers to associate with this record?		
Hint:	Fill in one ID number here and if there are additional ID numbers complete next question series		
		Yes	
		No	10002

ID	Question	Answer(s)	Alternate IDs
alt_id_1_1	Enter Alternate Id (1)		
Hint:			
		DSS_ID	
		Alternate_ID	

ID	Question	Answer(s)	Skip
alt_id_2	Do you have another alternate identifier to associate with this record?		
Hint:			
		Yes	
		No	10002

ID	Question	Answer(s)	Alternate IDs
alt_id_2_2	Select the type of ID recorded		
Hint:			
		DSS_ID	
		Alternate_ID	

ID	Question	Answer(s)	Skip
alt_id_3	Do you have another alternate identifier to associate with this record?		
Hint:			
		Yes	
		No	10002

ID	Question	Answer(s)	Alternate IDs
alt_id_3_2	Select the type of ID recorded		
Hint:			
		DSS_ID	
		Alternate_ID	

ID	Question	Answer(s)	Skip
presets	Information about the prevalence of malaria and HIV. This section is for the interviewer to complete and is not to be asked of the respondents.		
Hint:			

ID	Question	Answer(s)	Skip
Id10002	[Is this a region of high HIV/AIDS prevalence?]		
Hint:	Should be completed by the central office. HIGH corresponds to >1% of deaths, LOW around 0.1%, VERY LOW < 0.01%.		
		☐ High	
		☐ Low	
		☐ Very low	

ID	Question	Answer(s)	Skip
Id10003	[Is this a region of high malaria prevalence?]		
Hint:	Should be completed by the central office. HIGH corresponds to > 1% of deaths, LOW around 0.1%, VERY LOW < 0.01%.		
		☐ High	
		☐ Low	
		☐ Very low	

ID	Question	Answer(s)	Skip
Id10004	[During which season did (s)he die?]		
Hint:			
		☐ Wet	
		☐ Dry	
		☐ Doesn't know	

ID	Question	Answer(s)	Skip
Id10007	What is the name of VA respondent?		
Hint:			

ID	Question	Answer(s)	Skip
Id10008	What is your/the respondent's relationship to the deceased?		
Hint:	First verify if the respondent is a family member, and only if it is not a family member choose the other categories like health worker or public official.		
		☐ Parent	
		☐ Child	
		☐ Other family member	
		☐ Friend	
		☐ Health worker	
		☐ Public official	
		☐ Another relationship	

ID	Question	Answer(s)	Skip
Id10009	Did you/the respondent live with the deceased in the period leading to her/his death?		
Hint:			
		Yes	
		No	
		Doesn't know	
		Refused to answer	

ID	Question	Answer(s)	Skip
Id10010	[Name of VA interviewer]		
Hint:			

ID	Question	Answer(s)	Skip
Id10011	[Time at start of interview]		
Hint:	[hh:mm 24h]		
		HH: MM:	

ID	Question	Answer(s)	Skip
Id10012	[Date of interview]		
Hint:	[dd/mm/yyyy]		
		Day/ Month/ Year/	

ID	Question	Answer(s)	Skip
Id10013	[Did the respondent give consent?]		
Hint:			
		Yes	
		No	10481

ID	Question	Answer(s)	Skip
Id10017	What was the first or given name(s) of the deceased?		
Hint:			

ID	Question	Answer(s)	Skip
Id10018	What was the surname (or family name) of the deceased?		
Hint:			

ID	Question	Answer(s)	Skip
Id10019	What was the sex of the deceased?		
Hint:			
		Female	
		Male	

ID	Question	Answer(s)	Skip
Id10020	Is the date of birth known?		
Hint:			
		Yes	
		No	10022
		Refused to answer	10022

ID	Question	Answer(s)	Skip
Id10021	When was the deceased born?		
Hint:	Enter [Day/Month/Year] to select previous years, click or tap the month name, then click or tap the year.		
		Day/ Month/ Year/	

ID	Question	Answer(s)	Skip
Id10022	Is the date of death known?		
Hint:			
		Yes	
		No	10024
		Refused to answer	Age_group

ID	Question	Answer(s)	Skip
Id10023	When did (s)he die?		
Hint:	Enter [Day/Month/Year]		
		Day/ Month/ Year/	

ID	Question	Answer(s)	Skip
Id10024	Please indicate the year of death.		
Hint:			
		Year:	

ID	Question	Answer(s)	Skip
age_group	What age group corresponds to the deceased?		
Hint:	(1) Neonatal 0-27 completed days; (2) Child 28 days - 11 years; (3) Adult - above 11 years		
		Neonate	

ID	Question	Answer(s)	Skip
age_neonate_u	[Enter neonate's age in]:		
Hint:	If < 1 day, enter in hours or minutes. If < 1 hour, enter in minutes.		
		Days	
		Hours	
		Minutes	

ID	Question	Answer(s)	Skip
Id10058	Where did the deceased die?		
Hint:			
		☐ Hospital	
		☐ Other health facility	
		☐ Home	
		☐ On route to hospital or facility	
		☐ Other	
		☐ Doesn't know	
		☐ Refused to answer	

ID	Question	Answer(s)	Skip
Id10051	[Is there a need to collect civil registration data on the deceased?]		
Hint:	If you choose 'No', this question allows you to skip asking details about place of residence, education and family. The question on marriage status will always be asked for adults.		
		☐ Yes	
		☐ No	10069

ID	Question	Answer(s)	Skip
Id10052	What was her/his citizenship/nationality?		
Hint:			
		☐ Citizen at birth	
		☐ Naturalized citizen	
		☐ Foreign national	
		☐ Doesn't know	

ID	Question	Answer(s)	Skip
Id10053	What was her/his ethnicity?		
Hint:	Enter a “-“ if this information is not available.		

ID	Question	Answer(s)	Skip
Id10054	What was her/his place of birth?		
Hint:	Specify here village and district, a question on the facility and circumstances will be asked later. Enter a “-“ if this information is not available.		

ID	Question	Answer(s)	Skip
Id10055	What was her/his place of usual residence? (the place where the person lived most of the year)		
Hint:	For perinatal cases, just ask for the address of the health facility or if released at home, the home address.		

ID	Question	Answer(s)	Skip
Id10057	Where did the death occur? (specify country, province, district, village)		
Hint:			

ID	Question	Answer(s)	Skip
Id10061	What was the name of the father?		
Hint:			

ID	Question	Answer(s)	Skip
Id10062	What was the name of the mother?		
Hint:			

ID	Question	Answer(s)	Skip
Id10069	[Is there a need to collect civil registration numbers on the deceased?]		
Hint:	If "Yes" ask to see a certificate.		
		Yes	
		No	10077

ID	Question	Answer(s)	Skip
Id10070	[Death registration number/certificate]		
Hint:	Enter a "-" if this information is not available.		

ID	Question	Answer(s)	Skip
Id10071	[Date of registration]		
Hint:	If date unknown, enter "January 1, 2000" to indicate not available.		
		Day/ Month/ Year/	

ID	Question	Answer(s)	Skip
Id10072	[Place of registration]		
Hint:	Enter a "-" if this information is not available.		

ID	Question	Answer(s)	Skip
Id10073	[National identification number of deceased]		
Hint:	Enter a "-" if this information is not available. For children and newborns that have no ID number, use the mother's ID. If mother's ID is not available, use the father's ID.		

ID	Question	Answer(s)	Skip
Id10077	Did (s)he suffer from any injury or accident that led to her/his death?		
Hint:			
		Yes	
		No	10104
		Doesn't know	10104
		Refused to answer	10104

ID	Question	Answer(s)	Skip
Id10079	Was it a road traffic accident?		
Hint:			
		Yes	
		No	10082
		Doesn't know	10082
		Refused to answer	10082

ID	Question	Answer(s)	Skip
Id10080	What was her/his role in the road traffic accident?		
Hint:			
		Pedestrian	
		Driver or passenger in car or light vehicle	
		Driver or passenger in bus or heavy vehicle	
		Driver or passenger on a motorcycle	
		Driver or passenger on a pedal cycle	
		Other	

ID	Question	Answer(s)	Skip
Id10081	What was the counterpart that was hit during the road traffic accident?		
Hint:			
		Pedestrian	10098
		Stationary object	10098
		Car or light vehicle	10098
		Bus or heavy vehicle	10098
		Motorcycle	10098
		Pedal cycle	10098
		Other	10098

ID	Question	Answer(s)	Skip
Id10082	Was (s)he injured in a non-road traffic accident?		
Hint:			
		☐ Yes	☐
		☐ No	☐
		☐ Doesn't know	☐
		☐ Refused to answer	☐

ID	Question	Answer(s)	Skip
Id10083	Was (s)he injured in a fall?		
Hint:			
		☐ Yes	☐
		☐ No	☐
		☐ Doesn't know	☐
		☐ Refused to answer	☐

ID	Question	Answer(s)	Skip
Id10084	Was there any poisoning?		
Hint:	This includes accidents and cases where it is unknown if it was an accident or whether there was intentional violence.		
		☐ Yes	☐
		☐ No	☐
		☐ Doesn't know	☐
		☐ Refused to answer	☐

ID	Question	Answer(s)	Skip
Id10085	Did (s)he die of drowning?		
Hint:			
		☐ Yes	☐
		☐ No	☐
		☐ Doesn't know	☐
		☐ Refused to answer	☐

ID	Question	Answer(s)	Skip
Id10086	Was (s)he injured by a bite or sting by venomous animal?		
Hint:	This includes accidents and cases where it is unknown if it was an accident or whether there was intentional violence.		
		Yes	10088
		No	
		Doesn't know	
		Refused to answer	

ID	Question	Answer(s)	Skip
Id10087	Was (s)he injured by an animal or insect (non-venomous)?		
Hint:			
		Yes	
		No	10089
		Doesn't know	10089
		Refused to answer	10089

ID	Question	Answer(s)	Skip
Id10088	What was the animal/insect?		
Hint:			
		Dog	
		Snake	
		Insect or scorpion	
		Other	
		Doesn't know	

ID	Question	Answer(s)	Skip
Id10089	Was (s)he injured by burns/fire?		
Hint:			
		Yes	
		No	
		Doesn't know	
		Refused to answer	

ID	Question	Answer(s)	Skip
Id10090	Was (s)he subject to violence (suicide, homicide, abuse)?		
Hint:	Don't say suicide for under -10-year olds.		
		Yes	
		No	
		Doesn't know	
		Refused to answer	

ID	Question	Answer(s)	Skip
Id10091	Was (s)he injured by a firearm?		
Hint:			
		Yes	
		No	
		Doesn't know	
		Refused to answer	

ID	Question	Answer(s)	Skip
Id10092	Was (s)he stabbed, cut or pierced?		
Hint:			
		Yes	
		No	
		Doesn't know	
		Refused to answer	

ID	Question	Answer(s)	Skip
Id10093	Was (s)he strangled?		
Hint:			
		Yes	
		No	
		Doesn't know	
		Refused to answer	

ID	Question	Answer(s)	Skip
Id10094	Was (s)he injured by a blunt force?		
Hint:			
		Yes	
		No	
		Doesn't know	
		Refused to answer	

ID	Question	Answer(s)	Skip
Id10095	Was (s)he injured by a force of nature?		
Hint:			
		Yes	
		No	
		Doesn't know	
		Refused to answer	

ID	Question	Answer(s)	Skip
Id10096	Was it electrocution?		
Hint:	This includes accidents and cases where it is unknown if it was an accident or whether there was intentional violence.		
		Yes	
		No	
		Doesn't know	
		Refused to answer	

ID	Question	Answer(s)	Skip
Id10097	Did (s)he encounter any other injury?		
Hint:			
		Yes	
		No	
		Doesn't know	
		Refused to answer	

ID	Question	Answer(s)	Skip
Id10098	Was the injury accidental?		
Hint:			
		Yes	
		No	
		Doesn't know	
		Refused to answer	

ID	Question	Answer(s)	Skip
Id10100	Was the injury or accident intentionally inflicted by someone else?		
Hint:			
		Yes	
		No	
		Doesn't know	
		Refused to answer	

ID	Question	Answer(s)	Skip
Id10104	Did the baby ever cry?		
Hint:			
		Yes	
		No	10109
		Doesn't know	10109
		Refused to answer	10109

ID	Question	Answer(s)	Skip
Id10105	Did the baby cry immediately after birth, even if only a little bit?		
Hint:			
		Yes	
		No	
		Doesn't know	
		Refused to answer	

ID	Question	Answer(s)	Skip
Id10106	How many minutes after birth did the baby first cry?		
Hint:			
		Minutes	

ID	Question	Answer(s)	Skip
Id10107	Did the baby stop being able to cry?		
Hint:			
		Yes	
		No	10109
		Doesn't know	10109
		Refused to answer	10109

ID	Question	Answer(s)	Skip
Id10108	How many hours before death did the baby stop crying?		
Hint:	60 Minutes=1 Hour; If < 1 Hour, Record "0" Hours.		
		Hours	

ID	Question	Answer(s)	Skip
Id10109	Did the baby ever move?		
Hint:			
		Yes	
		No	
		Doesn't know	
		Refused to answer	

ID	Question	Answer(s)	Skip
Id10110	Did the baby ever breathe?		
Hint:			
		Yes	
		No	10114
		Doesn't know	10114
		Refused to answer	10114

ID	Question	Answer(s)	Skip
Id10111	Did the baby breathe immediately after birth, even a little?		
Hint:			
		Yes	
		No	
		Doesn't know	
		Refused to answer	

ID	Question	Answer(s)	Skip
Id10112	Did the baby have a breathing problem?		
Hint:			
		Yes	
		No	
		Doesn't know	
		Refused to answer	

ID	Question	Answer(s)	Skip
Id10113	Was the baby given assistance to breathe at birth?		
Hint:			
		Yes	
		No	
		Doesn't know	
		Refused to answer	

ID	Question	Answer(s)	Skip
Id10114	If the baby didn't show any sign of life, was it born dead?		
Hint:	This question serves to finally determine if the baby was born alive or dead.		
		Yes	Still birth
		No	Live birth
		Doesn't know	
		Refused to answer	

ID	Question	Answer(s)	Skip
Id10115	Were there any bruises or signs of injury on baby's body after the birth?		
Hint:			
		☐ Yes	
		☐ No	
		☐ Doesn't know	
		☐ Refused to answer	

ID	Question	Answer(s)	Skip
Id10116	Was the baby’s body soft, pulpy and discoloured and the skin peeling away?		
Hint:	Macerated means the body was pulpy. This indicates that the baby has been dead inside the mother for some time.		
		☐ Yes	10347
		☐ No	10347
		☐ Doesn't know	10347
		☐ Refused to answer	10347

ID	Question	Answer(s)	Skip
Id10351	How old was the baby when the fatal illness started?		
Hint:	The answer could be in months or days, but for data entry convert in days. Less than 24 hours = 0 days.		
		☐ Days ☐☐	

ID	Question	Answer(s)	Skip
Id10408	Before the illness that led to death, was the baby/the child growing normally?		
Hint:			
		☐ Yes	
		☐ No	
		☐ Doesn't know	
		☐ Refused to answer	

ID	Question	Answer(s)	Skip
illness_length	For how long was (s)he ill before death? Enter length of illness in:		
Hint:			

ID	Question	Answer(s)	Skip
Id10120	Enter length of illness in days:		
Hint:	Less than 24 hours = 0 days. If more than 28 days, go back and enter duration in months (if not neonate).		
		☐ Days ☐☐	

ID	Question	Answer(s)	Skip
Id10122	Enter length of illness in weeks:		
Hint:	If duration given is more then 4 weeks, need to verify again age at death if neonate.		
		☐ Weeks ☐☐	

ID	Question	Answer(s)	Skip
Id10121	**Enter length of illness in month:**		
Hint:	Not applicable for newborn. If duration given is more then one month, need to verify again age at death.		
		Months	

ID	Question	Answer(s)	Skip
Id10123	**Did (s)he die suddenly?**		
Hint:	Suddenly means died unexpectedly within 24 hours of being in regular health.		
		Yes	
		No	
		Doesn't know	
		Refused to answer	

ID	Question	Answer(s)	Skip
Id10147	**Did (s)he have a fever?**		
Hint:			
		Yes	
		No	10153
		Doesn't know	10153
		Refused to answer	10153

ID	Question	Answer(s)	Skip
Id10148	**How many days did the fever last?**		
Hint:	Less than 1 day = "0". Use 1 week = 7 days to determine the number of days.		
		Days	

ID	Question	Answer(s)	Skip
Id10149	**Did the fever continue until death?**		
Hint:			
		Yes	
		No	
		Doesn't know	
		Refused to answer	

ID	Question	Answer(s)	Skip
Id10153	**Did (s)he have a cough?**		
Hint:			
		Yes	
		No	10159
		Doesn't know	10159
		Refused to answer	10159

ID	Question	Answer(s)	Skip
Id10158	Did (s)he make a whooping sound when coughing?		
Hint:			
		Yes	
		No	
		Doesn't know	
		Refused to answer	

ID	Question	Answer(s)	Skip
Id10159	Did (s)he have any difficulty breathing?		
Hint:			
		Yes	
		No	10166
		Doesn't know	10166
		Refused to answer	10166

ID	Question	Answer(s)	Skip
Id10161_1	For how many days did the difficulty breathing last?		
Hint:			
		Days	

ID	Question	Answer(s)	Skip
Id10166	During the illness that led to death, did (s)he have fast breathing?		
Hint:			
		Yes	
		No	10168
		Doesn't know	10168
		Refused to answer	10168

ID	Question	Answer(s)	Skip
Id10167	For how many days did the fast breathing last?		
Hint:	Less than 1 day = “0”. Use 1 week = 7 days to determine the number of days.		
		Days	

ID	Question	Answer(s)	Skip
Id10168	Did (s)he have breathlessness?		
Hint:			
		Yes	
		No	10172
		Doesn't know	10172
		Refused to answer	10172

ID	Question	Answer(s)	Skip
Id10169	**For how many days did (s)he have breathlessness?**		
Hint:	Less than 1 day = "0." Use 1 week = 7 days to determine the number of days.		
		☐ Days	

ID	Question	Answer(s)	Skip
Id10172	**Did you see the lower chest wall/ribs being pulled in as the child breathed in?**		
Hint:	Ask for children under the age of 12 years. Show photos/video (if available).		
		☐ Yes	
		☐ No	
		☐ Doesn't know	
		☐ Refused to answer	

ID	Question	Answer(s)	Skip
Id10173	**During the illness that led to death did his/her breathing sound like any of the following:**		
Hint:	In case of adults demonstrate wheezing sound only because stridor and grunting is extremely rare in adults. Ask about wheezing, stridor and grunting only for children under the age of 12 years. Demonstrate each sound, play audio (if available), if in doubt select multiple.		
		☐ Stridor	
		☐ Grunting	
		☐ Wheezing	
		☐ No	
		☐ Doesn't know	
		☐ Refused to answer	

ID	Question	Answer(s)	Skip
Id10181	**Did (s)he have more frequent loose or liquid stools than usual?**		
Hint:	Ask the respondent about his/her understanding of what is diarrhoea (having more frequent loose or liquid stools than usual); if unclear or wrong, explain to the respondent what is diarrhoea.		
		☐ Yes	
		☐ No	10188
		☐ Doesn't know	10188
		☐ Refused to answer	10188

ID	Question	Answer(s)	Skip
Id10183	**How many stools did the baby or child have on the day that loose liquid stools were most frequent?**		
Hint:	Enter "99" if this information is not known.		
		Number of stools:	

ID	Question	Answer(s)	Skip
Id10184	How many days before death did the frequent loose or liquid stools start?		
Hint:	Less than 1 day= “0”. Use 1 week = 7 days to determine the number of days. Enter “99“ if this information is not known.		
		Days	

ID	Question	Answer(s)	Skip
Id10186	At any time during the final illness was there blood in the stools?		
Hint:			
		Yes	
		No	
		Doesn't know	
		Refused to answer	

ID	Question	Answer(s)	Skip
Id10188	Did (s)he vomit?		
Hint:			
		Yes	
		No	
		Doesn't know	
		Refused to answer	

ID	Question	Answer(s)	Skip
Id10189	Did (s)he vomit in the week preceding the death?		
Hint:			
		Yes	
		No	
		Doesn't know	
		Refused to answer	

ID	Question	Answer(s)	Skip
Id10214	Was (s)he unconscious during the illness that lead to death?		
Hint:			
		Yes	
		No	10219
		Doesn't know	10219
		Refused to answer	10219

ID	Question	Answer(s)	Skip
Id10215	Was (s)he unconscious for more than 24 hours before death?		
Hint:			
		☐ Yes	
		☐ No	
		☐ Doesn't know	
		☐ Refused to answer	

ID	Question	Answer(s)	Skip
Id10219	Did (s)he have convulsions?		
Hint:			
		☐ Yes	
		☐ No	
		☐ Doesn't know	
		☐ Refused to answer	

ID	Question	Answer(s)	Skip
Id10233	During the illness that led to death, did (s)he have any skin rash?		
Hint:			
		☐ Yes	
		☐ No	
		☐ Doesn't know	
		☐ Refused to answer	

ID	Question	Answer(s)	Skip
Id10239	During the illness that led to death, did he/she have areas of the skin that turned black?		
Hint:			
		☐ Yes	
		☐ No	
		☐ Doesn't know	
		☐ Refused to answer	

ID	Question	Answer(s)	Skip
Id10240	During the illness that led to death, did he/she have areas of the skin with redness and swelling?		
Hint:			
		Yes	
		No	
		Doesn't know	
		Refused to answer	

ID	Question	Answer(s)	Skip
Id10241	During the illness that led to death, did (s)he bleed from anywhere?		
Hint:			
		Yes	
		No	
		Doesn't know	
		Refused to answer	

ID	Question	Answer(s)	Skip
Id10265	Did (s)he have yellow discoloration of the eyes?		
Hint:			
		Yes	
		No	
		Doesn't know	
		Refused to answer	

ID	Question	Answer(s)	Skip
Id10271	Was the baby able to suckle or bottle-feed within the first 24 hours after birth?		
Hint:	Ask only if the child was < 1 year old when it died.		
		Yes	
		No	
		Doesn't know	
		Refused to answer	

ID	Question	Answer(s)	Skip
Id10272	**Did the baby ever suckle in a normal way?**		
Hint:	Ask only if the child was < 1 year old when it died.		
		Yes	
		No	
		Doesn't know	
		Refused to answer	

ID	Question	Answer(s)	Skip
Id10273	**Did the baby stop suckling?**		
Hint:	Ask only if the child was < 1 year old when it died.		
		Yes	
		No	10275
		Doesn't know	10275
		Refused to answer	10275

ID	Question	Answer(s)	Skip
Id10274	**How many days after birth did the baby stop suckling?**		
Hint:	Ask only if the child was < 1 year old when it died.		
		Days	

ID	Question	Answer(s)	Skip
Id10275	**Did the baby have convulsions starting within the first 24 hours of life?**		
Hint:	Ask only if the child was < 1 year old when it died.		
		Yes	10277
		No	
		Doesn't know	
		Refused to answer	

ID	Question	Answer(s)	Skip
Id10276	**Did the baby have convulsions starting more than 24 hours after birth?**		
Hint:	Ask only if the child was < 1 year old when it died.		
		Yes	
		No	
		Doesn't know	
		Refused to answer	

ID	Question	Answer(s)	Skip
Id10277	**Did the baby's body become stiff, with the back arched backwards?**		
Hint:	Ask only if the child was < 1 year old when it died.		
		Yes	
		No	
		Doesn't know	
		Refused to answer	

ID	Question	Answer(s)	Skip
Id10278	**During the illness that led to death, did the baby have a bulging or raised fontanelle? (ask only up to 18 months)**		
Hint:	Show photo (if available)		
		Yes	10281
		No	
		Doesn't know	
		Refused to answer	

ID	Question	Answer(s)	Skip
Id10279	**During the illness that led to death, did the baby have a sunken fontanelle? (ask only up to 18 months)**		
Hint:	Show photo (if available)		
		Yes	
		No	
		Doesn't know	
		Refused to answer	

ID	Question	Answer(s)	Skip
Id10281	**During the illness that led to death, did the baby become unresponsive or unconscious?**		
Hint:			
		Yes	
		No	10284
		Doesn't know	10284
		Refused to answer	10284

ID	Question	Answer(s)	Skip
Id10282	Did the baby become unresponsive or unconscious soon after birth, within less than 24 hours?		
Hint:			
		Yes	
		No	
		Doesn't know	
		Refused to answer	

ID	Question	Answer(s)	Skip
Id10283	Did the baby become unresponsive or unconscious more than 24 hours after birth?		
Hint:			
		Yes	
		No	
		Doesn't know	
		Refused to answer	

ID	Question	Answer(s)	Skip
Id10284	During the illness that led to death, did the baby become cold to touch?		
Hint:			
		Yes	
		No	10286
		Doesn't know	10286
		Refused to answer	10286

ID	Question	Answer(s)	Skip
Id10285	How many days old was the baby when it started feeling cold to touch?		
Hint:	Less than 1 day = “0.” Use 1 week = 7 days to determine the number of days.		
		Days	

ID	Question	Answer(s)	Skip
Id10286	During the illness that led to death, did the baby become lethargic after a period of normal activity?		
Hint:			
		Yes	
		No	
		Doesn't know	
		Refused to answer	

ID	Question	Answer(s)	Skip
Id10287	Did the baby have redness or pus drainage from the umbilical cord stump?		
Hint:			
		Yes	
		No	
		Doesn't know	
		Refused to answer	

ID	Question	Answer(s)	Skip
Id10288	During the illness that led to death, did the baby have skin ulcer(s) or pits?		
Hint:			
		Yes	
		No	
		Doesn't know	
		Refused to answer	

ID	Question	Answer(s)	Skip
Id10289	During the illness that led to death, did the baby have yellow skin, palms (hand) or soles (foot)?		
Hint:			
		Yes	
		No	
		Doesn't know	
		Refused to answer	

ID	Question	Answer(s)	Skip
Id10290	Did the baby or infant appear to be healthy and then just die suddenly?		
Hint:	Suddenly means died unexpectedly within 24 hours of being in regular health.		
		Yes	
		No	
		Doesn't know	
		Refused to answer	

ID	Question	Answer(s)	Skip
Id10347	Was the baby born more than one month early?		
Hint:			
		Yes	
		No	
		Doesn't know	
		Refused to answer	

ID	Question	Answer(s)	Skip
Id10354	**Was the child part of a multiple birth?**		
Hint:	If two or more children are born at the same time, it is counted as a multiple birth, even if one or more of the babies are born dead.		
		Yes	
		No	10356
		Doesn't know	10356
		Refused to answer	10356

ID	Question	Answer(s)	Skip
Id10355	**Was the child the first, second, or later in the birth order?**		
Hint:			
		First	
		Second or later	

ID	Question	Answer(s)	Skip
Id10356	**Is the mother still alive?**		
Hint:	If mother is present at the interview, select 'yes' without asking the question aloud. Only read this question if the respondent is not the mother and if it is not yet known if the mother is alive.		
		Yes	10360
		No	
		Doesn't know	
		Refused to answer	

ID	Question	Answer(s)	Skip
Id10357	**Did the mother die during or after the delivery?**		
Hint:			
		During delivery	10360
		After delivery	

ID	Question	Answer(s)	Skip
mother_death	**How long after the delivery did the mother die?**		
Hint:	The respondent may reply in months or days. If less than 24 hours, record "0" days. If < 1 month, record in days; if less than < 2 months, record in weeks; if ≥ 2 months, record in completed months.		
		Months	
		Weeks	
		Days	

ID	Question	Answer(s)	Skip
Id10358_1	**How many weeks after the delivery did the mother die?**		
Hint:	The respondent may reply in months or days. If less than 24 hours, record "0" days. If < 1 month, record in days; if less than < 2 months, record in weeks; if ≥ 2 months, record in completed months		
		Weeks	

ID	Question	Answer(s)	Skip
Id10360	**Where was the deceased born?**		
Hint:	Read the question and slowly read the first 5 choices. Respondent should hear all 5 choices and then respond.		
		Hospital	
		Other health facility	
		Home	
		On route to hospital or facility	
		Other	
		Doesn't know	
		Refused to answer	

ID	Question	Answer(s)	Skip
Id10361	**Did you/the mother receive professional assistance during the delivery? (ask only up to 1 year)**		
Hint:	Explain to the respondent what is meant by professional assistance: delivery attended by a medical professional (doctor, nurse or midwife).		
		Yes	
		No	
		Doesn't know	
		Refused to answer	

ID	Question	Answer(s)	Skip
Id10362	**At birth, was the baby of usual size?**		
Hint:	Show photos (if available), explain to the respondent that even if the answer is "no" some more questions will be asked, just to make sure no important detail has been missed.		
		Yes	10366
		No	
		Doesn't know	
		Refused to answer	

ID	Question	Answer(s)	Skip
Id10363	**At birth, was the baby smaller than usual, (weighing under 2.5 kg)?**		
Hint:	Show photos (if available).		
		Yes	
		No	10365
		Doesn't know	10365
		Refused to answer	10365

ID	Question	Answer(s)	Skip
Id10364	At birth, was the baby very much smaller than usual, (weighing under 1 kg)?		
Hint:	Show photos (if available).		
		Yes	10366
		No	10366
		Doesn't know	10366
		Refused to answer	10366

ID	Question	Answer(s)	Skip
Id10365	At birth, was the baby larger than usual, (weighing over 4.5 kg)?		
Hint:	Show photos (if available).		
		Yes	
		No	
		Doesn't know	
		Refused to answer	

ID	Question	Answer(s)	Skip
Id10366	What was the weight (in grammes) of the deceased at birth?		
Hint:	Respondents may give the answer in kilograms. For the data entry, convert to grammes. 1 kilogram=1,000 grammes. Enter "9999" for "Don't know." Enter "8888" for "Refused to answer."		
		Grammes:	

ID	Question	Answer(s)	Skip
Id10367	How many months long was the pregnancy before the child was born? (ask only up to 1 year)		
Hint:	If don't know enter "99"		
		Months	

ID	Question	Answer(s)	Skip
Id10368	Were there any complications in the late part of the pregnancy (defined as the last 3 months, before labour)? (ask only up to 1 year)		
Hint:			
		Yes	
		No	
		Doesn't know	
		Refused to answer	

ID	Question	Answer(s)	Skip
Id10369	Were there any complications during labour or delivery? (ask only up to 1 year)		
Hint:			
		Yes	
		No	
		Doesn't know	
		Refused to answer	

ID	Question	Answer(s)	Skip
Id10370	Was any part of the baby physically abnormal at time of delivery? (for example: body part too large or too small, additional growth on body)?		
Hint:			
		Yes	
		No	10376
		Doesn't know	
		Refused to answer	

ID	Question	Answer(s)	Skip
Id10371	Did the baby/ child have a swelling or defect on the back at time of birth?		
Hint:			
		Yes	
		No	
		Doesn't know	
		Refused to answer	

ID	Question	Answer(s)	Skip
Id10372	Did the baby/ child have a very large head at time of birth?		
Hint:			
		Yes	10376
		No	
		Doesn't know	
		Refused to answer	

ID	Question	Answer(s)	Skip
Id10373	Did the baby/ child have a very small head at time of birth?		
Hint:			
		Yes	
		No	
		Doesn't know	
		Refused to answer	

ID	Question	Answer(s)	Skip
Id10376	Was the baby moving in the last few days before the birth?		
Hint:			
		Yes	
		No	
		Doesn't know	
		Refused to answer	

ID	Question	Answer(s)	Skip
Id10377	Did the baby stop moving in the womb before labour started?		
Hint:			
		Yes	
		No	10382
		Doesn't know	
		Refused to answer	

ID	Question	Answer(s)	Skip
last_time_move	When did the baby last move?”		
Hint:			
		Days	10379
		Hours	10380

ID	Question	Answer(s)	Skip
Id10379	How many days before labour did you or the mother last feel the baby move? (maybe the respondent or health worker had examined the mother)		
Hint:	Enter duration as appropriate in hours or days.		
		Days	10382

ID	Question	Answer(s)	Skip
Id10380	How many hours before labour did you or the mother last feel the baby move? (maybe the respondent or health worker had examined the mother)		
Hint:	Enter duration as appropriate in hours or days. If < 24 hours, record "0".		
		Hours	10382

ID	Question	Answer(s)	Skip
Id10382	How many hours did labour and delivery take?		
Hint:	If less than one hour enter "0".		
		Hours	

ID	Question	Answer(s)	Skip
Id10383	Was the baby born 24 hours or more after the water broke?		
Hint:			
		Yes	
		No	
		Doesn't know	
		Refused to answer	

ID	Question	Answer(s)	Skip
Id10384	Was the liquor foul smelling?		
Hint:			
		Yes	
		No	
		Doesn't know	
		Refused to answer	

ID	Question	Answer(s)	Skip
Id10385	What was the colour of the liquor when the water broke?		
Hint:			
		Green or brown	
		Clear (normal)	
		Other	
		Doesn't know	
		Refused to answer	

ID	Question	Answer(s)	Skip
Id10387	Was the delivery normal vaginal, without forceps or vacuum?		
Hint:			
		Yes	10391
		No	
		Doesn't know	
		Refused to answer	

ID	Question	Answer(s)	Skip
Id10388	Was the delivery vaginal, with forceps or vacuum?		
Hint:			
		Yes	10391
		No	
		Doesn't know	
		Refused to answer	

ID	Question	Answer(s)	Skip
Id10389	Was the delivery a Caesarean section?		
Hint:			
		Yes	
		No	
		Doesn't know	
		Refused to answer	

ID	Question	Answer(s)	Skip
Id10391	Did you/the mother receive any vaccinations since reaching adulthood including during this pregnancy?		
Hint:			
		Yes	
		No	10394
		Doesn't know	10394
		Refused to answer	10394

ID	Question	Answer(s)	Skip
Id10392	How many doses?		
Hint:			
		Doses	

ID	Question	Answer(s)	Skip
Id10393	Did you/the mother receive tetanus toxoid (TT) vaccine?		
Hint:			
		Yes	
		No	
		Doesn't know	
		Refused to answer	

ID	Question	Answer(s)	Skip
Id10394	How many births, including stillbirths, did the baby's mother have before this baby?		
Hint:			
		Births	

ID	Question	Answer(s)	Skip
Id10395	During labour, did you/the baby's mother suffer from fever?		
Hint:			
		☐ Yes	
		☐ No	
		☐ Doesn't know	
		☐ Refused to answer	

ID	Question	Answer(s)	Skip
Id10396	During the last 3 months of pregnancy, labour or delivery, did the you/baby's mother suffer from high blood pressure?		
Hint:			
		☐ Yes	
		☐ No	
		☐ Doesn't know	
		☐ Refused to answer	

ID	Question	Answer(s)	Skip
Id10397	Did you/the baby's mother have diabetes mellitus?		
Hint:			
		☐ Yes	
		☐ No	
		☐ Doesn't know	
		☐ Refused to answer	

ID	Question	Answer(s)	Skip
Id10398	Did you/the baby's mother have foul smelling vaginal discharge during pregnancy or after delivery?		
Hint:			
		☐ Yes	
		☐ No	
		☐ Doesn't know	
		☐ Refused to answer	

ID	Question	Answer(s)	Skip
Id10399	During the last 3 months of pregnancy, labour or delivery, did you/the baby's mother suffer from convulsions?		
Hint:			
		☐ Yes	
		☐ No	
		☐ Doesn't know	
		☐ Refused to answer	

ID	Question	Answer(s)	Skip
Id10400	During the last 3 months of pregnancy did you/the baby's mother suffer from blurred vision?		
Hint:			
		☐ Yes	
		☐ No	
		☐ Doesn't know	
		☐ Refused to answer	

ID	Question	Answer(s)	Skip
Id10401	Did you/the baby's mother have severe anemia?		
Hint:			
		☐ Yes	
		☐ No	
		☐ Doesn't know	
		☐ Refused to answer	

ID	Question	Answer(s)	Skip
Id10402	Did you/the baby's mother have vaginal bleeding during the last 3 months of pregnancy but before labour started?		
Hint:			
		☐ Yes	
		☐ No	
		☐ Doesn't know	
		☐ Refused to answer	

ID	Question	Answer(s)	Skip
Id10403	Did the baby's bottom, feet, arm or hand come out of the vagina before its head?		
Hint:			
		Yes	
		No	
		Doesn't know	
		Refused to answer	

ID	Question	Answer(s)	Skip
Id10404	Was the umbilical cord wrapped more than once around the neck of the child at birth?		
Hint:			
		Yes	
		No	
		Doesn't know	
		Refused to answer	

ID	Question	Answer(s)	Skip
Id10405	Was the umbilical cord delivered first?		
Hint:			
		Yes	
		No	
		Doesn't know	
		Refused to answer	

ID	Question	Answer(s)	Skip
Id10406	Was the baby blue in colour at birth?		
Hint:			
		Yes	
		No	
		Doesn't know	
		Refused to answer	

ID	Question	Answer(s)	Skip
Id10418	Did (s)he receive any treatment for the illness that led to death?		
Hint:			
		Yes	
		No	10428
		Doesn't know	10428
		Refused to answer	10428

ID	Question	Answer(s)	Skip
Id10419	Did (s)he receive oral rehydration salts?		
Hint:			
		☐ Yes	
		☐ No	
		☐ Doesn't know	
		☐ Refused to answer	

ID	Question	Answer(s)	Skip
Id10420	Did (s)he receive (or need) intravenous fluids (drip) treatment?		
Hint:			
		☐ Yes	
		☐ No	
		☐ Doesn't know	
		☐ Refused to answer	

ID	Question	Answer(s)	Skip
Id10421	Did (s)he receive (or need) a blood transfusion?		
Hint:			
		☐ Yes	
		☐ No	
		☐ Doesn't know	
		☐ Refused to answer	

ID	Question	Answer(s)	Skip
Id10422	Did (s)he receive (or need) treatment/food through a tube passed through the nose?		
Hint:			
		☐ Yes	
		☐ No	
		☐ Doesn't know	
		☐ Refused to answer	

ID	Question	Answer(s)	Skip
Id10423	Did (s)he receive (or need) injectable antibiotics?		
Hint:			
		☐ Yes	
		☐ No	
		☐ Doesn't know	
		☐ Refused to answer	

ID	Question	Answer(s)	Skip
Id10424	Did (s)he receive (or need) antiretroviral therapy (ART)?		
Hint:			
		Yes	
		No	
		Doesn't know	
		Refused to answer	

ID	Question	Answer(s)	Skip
Id10425	Did (s)he have (or need) an operation for the illness?		
Hint:			
		Yes	
		No	
		Doesn't know	
		Refused to answer	

ID	Question	Answer(s)	Skip
Id10428	Had (s)he received immunizations?		
Hint:			
		Yes	
		No	10432
		Doesn't know	10432
		Refused to answer	10432

ID	Question	Answer(s)	Skip
Id10429	Do you have the child's vaccination card?		
Hint:			
		Yes	
		No	10432
		Doesn't know	10432
		Refused to answer	10432

ID	Question	Answer(s)	Skip
Id10430	Can I see the vaccination card (note the vaccines the child received)?		
Hint:	Only fill in the vaccination sheet if the respondent has the vaccination card at the present moment during the VA interview; the interviewer should fill in the vaccination sheet, not the respondent. If applicable, take photo with name covered.		
		Yes	
		No	10432
		Doesn't know	10432
		Refused to answer	10432

ID	Question	Answer(s)	Skip
Id10431	[Note vaccines here]		
Hint:	If applicable, record vaccine, date, and age of child		

ID	Question	Answer(s)	Skip
Id10432	Was care sought outside the home while (s)he had this illness?		
Hint:			
		Yes	
		No	10435
		Doesn't know	10435
		Refused to answer	10435

ID	Question	Answer(s)	Skip
Id10433	**Where or from whom did you seek care?**		
Hint:	Read the question and read the choices. Select all that apply.		
		☐ Traditional healer	
		☐ Homeopath	
		☐ Religious leader	
		☐ Government hospital	
		☐ Government health center or clinic	
		☐ Private hospital	
		☐ Community-based practitioner associated with health system	
		☐ Trained birth attendant	
		☐ Private physician	
		☐ Relative, friend (outside household)	
		☐ Pharmacy	
		☐ Doesn't know	
		☐ Refused to answer	

ID	Question	Answer(s)	Skip
Id10434	**Record the name and address of any hospital, health center or clinic where care was sought**		
Hint:	Record information provided.		

ID	Question	Answer(s)	Skip
Id10435	Did a health care worker tell you the cause of death?		
Hint:			
		Yes	
		No	10437
		Doesn't know	10437
		Refused to answer	10437

ID	Question	Answer(s)	Skip
Id10436	What did the health care worker say?		
Hint:			

ID	Question	Answer(s)	Skip
Id10437	Do you have any health records that belonged to the deceased?		
Hint:			
		Yes	
		No	10445
		Doesn't know	10445
		Refused to answer	10445

ID	Question	Answer(s)	Skip
Id10438	Can I see the health records?		
Hint:			
		Yes	
		No	10445
		Doesn't know	10445
		Refused to answer	10445

ID	Question	Answer(s)	Skip
Id10439	[Record the date of the most recent (last) visit]		
Hint:	If date unknown, record January 1, 2000 to indicate not available.		

Day/ Month/ Year/

ID	Question	Answer(s)	Skip
Id10445	Have you/has the deceased’s (biological) mother ever been tested for HIV?		
Hint:			
		Yes	
		No	
		Doesn't know	
		Refused to answer	

ID	Question	Answer(s)	Skip
Id10446	Have you/has the deceased’s (biological) mother ever been told she had HIV/AIDS by a health worker?		
Hint:			
		Yes	
		No	
		Doesn't know	
		Refused to answer	

ID	Question	Answer(s)	Skip
Id10450	In the final days before death, did s/he travel to a hospital or health facility?		
Hint:			
		Yes	
		No	10455
		Doesn't know	10455
		Refused to answer	10455

ID	Question	Answer(s)	Skip
Id10451	Did (s)he use motorised transport to get to the hospital or health facility?		
Hint:			
		Yes	
		No	
		Doesn't know	
		Refused to answer	

ID	Question	Answer(s)	Skip
Id10452	Were there any problems during admission to the hospital or health facility?		
Hint:			
		Yes	
		No	
		Doesn't know	
		Refused to answer	

ID	Question	Answer(s)	Skip
Id10453	Were there any problems with the way (s)he was treated (medical treatment, procedures, interpersonal attitudes, respect, dignity) in the hospital or health facility?		
Hint:			
		☐ Yes	
		☐ No	
		☐ Doesn't know	
		☐ Refused to answer	

ID	Question	Answer(s)	Skip
Id10454	Were there any problems getting medications or diagnostic tests in the hospital or health facility?		
Hint:			
		☐ Yes	
		☐ No	
		☐ Doesn't know	
		☐ Refused to answer	

ID	Question	Answer(s)	Skip
Id10455	Does it take more than 2 hours to get to the nearest hospital or health facility from the deceased's household?		
Hint:			
		☐ Yes	
		☐ No	
		☐ Doesn't know	
		☐ Refused to answer	

ID	Question	Answer(s)	Skip
Id10456	In the final days before death, were there any doubts about whether medical care was needed?		
Hint:			
		☐ Yes	
		☐ No	
		☐ Doesn't know	
		☐ Refused to answer	

ID	Question	Answer(s)	Skip
Id10457	In the final days before death, was traditional medicine used?		
Hint:			
		Yes	
		No	
		Doesn't know	
		Refused to answer	

ID	Question	Answer(s)	Skip
Id10458	In the final days before death, did anyone use a telephone or cell phone to call for help?		
Hint:			
		Yes	
		No	
		Doesn't know	
		Refused to answer	

ID	Question	Answer(s)	Skip
Id10459	Over the course of illness, did the total costs of care and treatment prohibit other household payments?		
Hint:			
		Yes	
		No	
		Doesn't know	
		Refused to answer	

ID	Question	Answer(s)	Skip
Id10462	Was a death certificate issued?		
Hint:	The following information serves only to complete cause of death information in some environments. In routine CRVS this information could be skipped in the interview, and information be collected from other sources, if available.		
		Yes	
		No	10476
		Doesn't know	10476
		Refused to answer	10476

ID	Question	Answer(s)	Skip
Id10463	**Can I see the death certificate?**		
Hint:	Fill in the following questions only if you are shown the copy of the certificate. Do not fill in just based on oral statements.		
		☐ Yes	
		☐ No	10476
		☐ Doesn't know	10476
		☐ Refused to answer	10476

ID	Question	Answer(s)	Skip
Id10464	**[Record the immediate cause of death from the certificate (line 1a)]**		
Hint:	An antecedent cause is the one that caused the one on the line above, e.g. diabetes mellitus may be an antecedent cause to kidney disease.		

ID	Question	Answer(s)	Skip
Id10465	**[Duration (Ia):]**		
Hint:			

ID	Question	Answer(s)	Skip
Id10466	**[Record the first antecedent cause of death from the certificate (line 1b)]**		
Hint:	For all following lines, add duration, if stated.		

ID	Question	Answer(s)	Skip
Id10467	**[Duration (Ib):]**		
Hint:			

ID	Question	Answer(s)	Skip
Id10468	**[Record the second antecedent cause of death from the certificate (line 1c)]**		
Hint:			

ID	Question	Answer(s)	Skip
Id10469	**[Duration (Ic):]**		
Hint:			

ID	Question	Answer(s)	Skip
Id10470	**[Record the third antecedent cause of death from the certificate (line 1d)]**		
Hint:			

ID	Question	Answer(s)	Skip
Id10471	**[Duration (Id):]**		
Hint:			

ID	Question	Answer(s)	Skip
Id10472	**[Record the contributing cause(s) of death from the certificate (part 2)]**		
Hint:			

ID	Question	Answer(s)	Skip
Id10473	**[Duration (part2):]**		
Hint:			

ID	Question	Answer(s)	Skip
Id10476	**NARRATIVE DESCRIPTION Thank you for your information. Now can you please tell me in your own words about the events that led to the death?**		
Hint:	AUDIO RECORDING • IF APPLICABLE, REQUEST CONSENT FOR AUDIO RECORDING: May I record your response? • IF YES, RECORD VERBAL CONSENT AT START OF RECORDING: “Do I have consent to record your response? WRITTEN NOTES AND PROBING • RECORD DETAILED NOTES OF RESPONSE; USE ADDITIONAL PAPER AS NEEDED. • IF NEEDED, PROBE FOR ADDL DETAILS ON WHEN RESPONDENT RECOGNIZED SYMPTOMS, CARE SOUGHT, BARRIERS TO CARE, ISSUES WITH TRANSPORT, ABNORMALITIES, ETC.		

ID	Question	Answer(s)	Skip
Id10479	Select any of the following words that were mentioned as present in the narrative.		
Hint:			
		Asphyxia	
		Incubator	
		Lung problem	
		Pneumonia	
		Preterm delivery	
		Respiratory distress	
		None of the above words were mentioned	
		Don't know	

ID	Question	Answer(s)	Skip
Id10481	Time at end of interview		
Hint:	hh:mm 24 h		
		HH: MM:	

ID	Question	Answer(s)	Skip
form_end	End of the Interview - Thanks		
Hint:			

CHAMPS Verbal Autopsy
Sample Questionnaire

Death of a child aged four weeks to 11 years

Powered by CHAMPS Engine

ID	Question	Answer(s)	Skip
alt_id_1	**Do you have any alternate identifiers to associate with this record?**		
Hint:	Fill in one ID number here and if there are additional ID numbers complete next question series		
		☐ Yes	
		☐ No	10002

ID	Question	Answer(s)	Alternate IDs
alt_id_1_1	**Enter Alternate Id (1)**		
Hint:			
		☐ DSS_ID	
		☐ Alternate_ID	

ID	Question	Answer(s)	Skip
alt_id_2	**Do you have another alternate identifier to associate with this record?**		
Hint:			
		☐ Yes	
		☐ No	10002

ID	Question	Answer(s)	Alternate IDs
alt_id_2_2	**Select the type of ID recorded**		
Hint:			
		☐ DSS_ID	
		☐ Alternate_ID	

ID	Question	Answer(s)	Skip
alt_id_3	**Do you have another alternate identifier to associate with this record?**		
Hint:			
		☐ Yes	
		☐ No	10002

ID	Question	Answer(s)	Alternate IDs
alt_id_3_2	**Select the type of ID recorded**		
Hint:			
		☐ DSS_ID	
		☐ Alternate_ID	

ID	Question	Answer(s)	Skip
presets	**Information about the prevalence of malaria and HIV. This section is for the interviewer to complete and is not to be asked of the respondents.**		
Hint:			

ID	Question	Answer(s)	Skip
Id10002	**[Is this a region of high HIV/AIDS prevalence?]**		
Hint:	Should be completed by the central office. HIGH corresponds to >1% of deaths, LOW around 0.1%, VERY LOW < 0.01%.		
		☐ High	
		☐ Low	
		☐ Very low	

ID	Question	Answer(s)	Skip
Id10003	[Is this a region of high malaria prevalence?]		
Hint:	Should be completed by the central office. HIGH corresponds to > 1% of deaths, LOW around 0.1%, VERY LOW < 0.01%.		
		☐ High	
		☐ Low	
		☐ Very low	

ID	Question	Answer(s)	Skip
Id10004	[During which season did (s)he die?]		
Hint:			
		☐ Wet	
		☐ Dry	
		☐ Doesn't know	

ID	Question	Answer(s)	Skip
Id10007	What is the name of VA respondent?		
Hint:			

ID	Question	Answer(s)	Skip
Id10008	What is your/the respondent's relationship to the deceased?		
Hint:	First verify if the respondent is a family member, and only if it is not a family member choose the other categories like health worker or public official.		
		☐ Parent	
		☐ Child	
		☐ Other family member	
		☐ Friend	
		☐ Health worker	
		☐ Public official	
		☐ Another relationship	

ID	Question	Answer(s)	Skip
Id10009	Did you/the respondent live with the deceased in the period leading to her/his death?		
Hint:			
		☐ Yes	
		☐ No	
		☐ Doesn't know	
		☐ Refused to answer	

ID	Question	Answer(s)	Skip
Id10010	[Name of VA interviewer]		
Hint:			

ID	Question	Answer(s)	Skip
Id10011	[Time at start of interview]		
Hint:	[hh:mm 24h]		
		HH: ☐ ☐ MM: ☐ ☐	

ID	Question	Answer(s)	Skip
Id10012	[Date of interview]		
Hint:	[dd/mm/yyyy]		
		Day/ Month/ Year/	

ID	Question	Answer(s)	Skip
Id10013	[Did the respondent give consent?]		
Hint:		Yes	
		No	10481

ID	Question	Answer(s)	Skip
Id10017	What was the first or given name(s) of the deceased?		
Hint:			

ID	Question	Answer(s)	Skip
Id10018	What was the surname (or family name) of the deceased?		
Hint:			

ID	Question	Answer(s)	Skip
Id10019	What was the sex of the deceased?		
Hint:		Female	
		Male	

ID	Question	Answer(s)	Skip
Id10020	Is the date of birth known?		
Hint:		Yes	
		No	10022
		Refused to answer	10022

ID	Question	Answer(s)	Skip
Id10021	When was the deceased born?		
Hint:	Enter [Day/Month/Year] to select previous years, click or tap the month name, then click or tap the year.		
		Day/ Month/ Year/	

ID	Question	Answer(s)	Skip
Id10022	Is the date of death known?		
Hint:		Yes	
		No	10024
		Refused to answer	Age_group

ID	Question	Answer(s)	Skip
Id10023	When did (s)he die?		
Hint:	Enter [Day/Month/Year]		
		Day/ Month/ Year/	

ID	Question	Answer(s)	Skip
Id10024	Please indicate the year of death.		
Hint:		Year:	

ID	Question	Answer(s)	Skip
age_group	What age group corresponds to the deceased?		
Hint:	(1) Neonatal 0-27 completed days; (2) Child 28 days - 11 years; (3) Adult - above 11 years		
		Child	

ID	Question	Answer(s)	Skip
age_child_unit	[Enter child's age in]:		
Hint:	Enter for one option only.		
		Days	
		Months	
		Years	

ID	Question	Answer(s)	Skip
Id10058	Where did the deceased die?		
Hint:			
		Hospital	
		Other health facility	
		Home	
		On route to hospital or facility	
		Other	
		Doesn't know	
		Refused to answer	

ID	Question	Answer(s)	Skip
Id10051	[Is there a need to collect civil registration data on the deceased?]		
Hint:	If you choose 'No', this question allows you to skip asking details about place of residence, education and family. The question on marriage status will always be asked for adults.		
		Yes	
		No	10069

ID	Question	Answer(s)	Skip
Id10052	What was her/his citizenship/nationality?		
Hint:			
		Citizen at birth	
		Naturalized citizen	
		Foreign national	
		Doesn't know	

ID	Question	Answer(s)	Skip
Id10053	What was her/his ethnicity?		
Hint:	Enter a “-“ if this information is not available.		

ID	Question	Answer(s)	Skip
Id10054	What was her/his place of birth?		
Hint:	Specify here village and district, a question on the facility and circumstances will be asked later. Enter a “-“ if this information is not available.		

ID	Question	Answer(s)	Skip
Id10055	What was her/his place of usual residence? (the place where the person lived most of the year)		
Hint:	For perinatal cases, just ask for the address of the health facility or if released at home, the home address.		

ID	Question	Answer(s)	Skip
Id10056	What was her/his place of normal residence 1 to 5 years before death?		
Hint:			

ID	Question	Answer(s)	Skip
Id10057	Where did the death occur? (specify country, province, district, village)		
Hint:			

ID	Question	Answer(s)	Skip
Id10061	What was the name of the father?		
Hint:			

ID	Question	Answer(s)	Skip
Id10062	What was the name of the mother?		
Hint:			

ID	Question	Answer(s)	Skip
Id10063	What was her/his highest level of schooling?		
Hint:	Mark "no formal education" if < 4 years old.		
		☐ No formal education	
		☐ Primary school	
		☐ Secondary school	
		☐ Higher than secondary school	
		☐ Doesn't know	
		☐ Refused to answer	

ID	Question	Answer(s)	Skip
Id10064	Was (s)he able to read and write?		
Hint:	Select 'Yes' also if only one of either reading or writing is known to the respondent. Mark "No" if < 6 years old.		
		☐ Yes	
		☐ No	
		☐ Doesn't know	
		☐ Refused to answer	

ID	Question	Answer(s)	Skip
Id10065	**What was her/his economic activity status in year prior to death?**		
Hint:	The deceased might have had several activities. Choose the one that was probably true for most of the year preceding illness and death. Mark "Other" if < 8 years old.		
		☐ Mainly unemployed	
		☐ Mainly employed	
		☐ Home-maker	
		☐ Pensioner	
		☐ Student	
		☐ Other	
		☐ Doesn't know	
		☐ Refused to answer	

ID	Question	Answer(s)	Skip
Id10066	**What was her/his occupation, that is, what kind of work did (s)he mainly do?**		
Hint:	If less than 8 years old, enter "-"for not applicable.		

ID	Question	Answer(s)	Skip
Id10069	**[Is there a need to collect civil registration numbers on the deceased?]**		
Hint:	If "Yes" ask to see a certificate.		
		☐ Yes	
		☐ No	10077

ID	Question	Answer(s)	Skip
Id10070	**[Death registration number/certificate]**		
Hint:	Enter a "-" if this information is not available.		

ID	Question	Answer(s)	Skip
Id10071	**[Date of registration]**		
Hint:	If date unknown, enter "January 1, 2000" to indicate not available.		
		Day/ Month/ Year/	

ID	Question	Answer(s)	Skip
Id10072	**[Place of registration]**		
Hint:	Enter a "-" if this information is not available.		

ID	Question	Answer(s)	Skip
Id10073	**[National identification number of deceased]**		
Hint:	Enter a "-" if this information is not available. For children and newborns that have no ID number, use the mother's ID. If mother's ID is not available, use the father's ID.		

ID	Question	Answer(s)	Skip
Id10077	Did (s)he suffer from any injury or accident that led to her/his death?		
Hint:		Yes	
		No	10120
		Doesn't know	10120
		Refused to answer	10120

ID	Question	Answer(s)	Skip
Id10079	Was it a road traffic accident?		
Hint:		Yes	
		No	10082
		Doesn't know	10082
		Refused to answer	10082

ID	Question	Answer(s)	Skip
Id10080	What was her/his role in the road traffic accident?		
Hint:		Pedestrian	
		Driver or passenger in car or light vehicle	
		Driver or passenger in bus or heavy vehicle	
		Driver or passenger on a motorcycle	
		Driver or passenger on a pedal cycle	
		Other	

ID	Question	Answer(s)	Skip
Id10081	What was the counterpart that was hit during the road traffic accident?		
Hint:		Pedestrian	10098
		Stationary object	10098
		Car or light vehicle	10098
		Bus or heavy vehicle	10098
		Motorcycle	10098
		Pedal cycle	10098
		Other	10098

ID	Question	Answer(s)	Skip
Id10082	Was (s)he injured in a non-road traffic accident?		
Hint:			
		Yes	
		No	
		Doesn't know	
		Refused to answer	

ID	Question	Answer(s)	Skip
Id10083	Was (s)he injured in a fall?		
Hint:			
		Yes	
		No	
		Doesn't know	
		Refused to answer	

ID	Question	Answer(s)	Skip
Id10084	Was there any poisoning?		
Hint:	This includes accidents and cases where it is unknown if it was an accident or whether there was intentional violence.		
		Yes	
		No	
		Doesn't know	
		Refused to answer	

ID	Question	Answer(s)	Skip
Id10085	Did (s)he die of drowning?		
Hint:			
		Yes	
		No	
		Doesn't know	
		Refused to answer	

ID	Question	Answer(s)	Skip
Id10086	Was (s)he injured by a bite or sting by venomous animal?		
Hint:	This includes accidents and cases where it is unknown if it was an accident or whether there was intentional violence.		
		Yes	10088
		No	
		Doesn't know	
		Refused to answer	

ID	Question	Answer(s)	Skip
Id10087	Was (s)he injured by an animal or insect (non-venomous)?		
Hint:			
		Yes	
		No	10089
		Doesn't know	10089
		Refused to answer	10089

ID	Question	Answer(s)	Skip
Id10088	What was the animal/insect?		
Hint:			
		Dog	
		Snake	
		Insect or scorpion	
		Other	
		Doesn't know	

ID	Question	Answer(s)	Skip
Id10089	Was (s)he injured by burns/fire?		
Hint:			
		Yes	
		No	
		Doesn't know	
		Refused to answer	

ID	Question	Answer(s)	Skip
Id10090	Was (s)he subject to violence (suicide, homicide, abuse)?		
Hint:	Don’t say suicide for under -10-year olds.		
		Yes	
		No	
		Doesn't know	
		Refused to answer	

ID	Question	Answer(s)	Skip
Id10091	Was (s)he injured by a firearm?		
Hint:			
		Yes	
		No	
		Doesn't know	
		Refused to answer	

ID	Question	Answer(s)	Skip
Id10092	Was (s)he stabbed, cut or pierced?		
Hint:			
		☐ Yes	
		☐ No	
		☐ Doesn't know	
		☐ Refused to answer	

ID	Question	Answer(s)	Skip
Id10093	Was (s)he strangled?		
Hint:			
		☐ Yes	
		☐ No	
		☐ Doesn't know	
		☐ Refused to answer	

ID	Question	Answer(s)	Skip
Id10094	Was (s)he injured by a blunt force?		
Hint:			
		☐ Yes	
		☐ No	
		☐ Doesn't know	
		☐ Refused to answer	

ID	Question	Answer(s)	Skip
Id10095	Was (s)he injured by a force of nature?		
Hint:			
		☐ Yes	
		☐ No	
		☐ Doesn't know	
		☐ Refused to answer	

ID	Question	Answer(s)	Skip
Id10096	Was it electrocution?		
Hint:	This includes accidents and cases where it is unknown if it was an accident or whether there was intentional violence.		
		☐ Yes	
		☐ No	
		☐ Doesn't know	
		☐ Refused to answer	

ID	Question	Answer(s)	Skip
Id10097	Did (s)he encounter any other injury?		
Hint:			
		Yes	
		No	
		Doesn't know	
		Refused to answer	

ID	Question	Answer(s)	Skip
Id10098	Was the injury accidental?		
Hint:			
		Yes	
		No	
		Doesn't know	
		Refused to answer	

ID	Question	Answer(s)	Skip
Id10100	Was the injury or accident intentionally inflicted by someone else?		
Hint:			
		Yes	
		No	
		Doesn't know	
		Refused to answer	

ID	Question	Answer(s)	Skip
illness_length	For how long was (s)he ill before death? Enter length of illness in:		
Hint:			

ID	Question	Answer(s)	Skip
Id10120	Enter length of illness in days:		
Hint:	Less than 24 hours = 0 days. If more than 28 days, go back and enter duration in months (if not neonate).		
		Days	

ID	Question	Answer(s)	Skip
Id10122	Enter length of illness in weeks:		
Hint:	If duration given is more then 4 weeks, need to verify again age at death if neonate.		
		Weeks	

ID	Question	Answer(s)	Skip
Id10121	Enter length of illness in month:		
Hint:	Not applicable for newborn. If duration given is more then one month, need to verify again age at death.		
		Months	

ID	Question	Answer(s)	Skip
Id10123	Did (s)he die suddenly?		
Hint:	Suddenly means died unexpectedly within 24 hours of being in regular health.		
		☐ Yes	
		☐ No	
		☐ Doesn't know	
		☐ Refused to answer	

ID	Question	Answer(s)	Skip
Id10125	Was there any diagnosis by a health professional of tuberculosis?		
Hint:	Remind the respondent that we are asking for the diagnosis assessed by a doctor, health worker, or other health professional during the final illness.		
		☐ Yes	
		☐ No	
		☐ Doesn't know	
		☐ Refused to answer	

ID	Question	Answer(s)	Skip
Id10126	Was an HIV test ever positive?		
Hint:			
		☐ Yes	
		☐ No	
		☐ Doesn't know	
		☐ Refused to answer	

ID	Question	Answer(s)	Skip
Id10127	Was there any diagnosis by a health professional of AIDS?		
Hint:	Remind the respondent that we are asking for the diagnosis assessed by a doctor, health worker, or other health professional during the final illness.		
		☐ Yes	
		☐ No	
		☐ Doesn't know	
		☐ Refused to answer	

ID	Question	Answer(s)	Skip
Id10128	Did (s)he have a recent positive test by a health professional for malaria?		
Hint:	Remind the respondent that we are asking for the diagnosis assessed by a doctor, health worker, or other health professional during the final illness.		
		☐ Yes	10130
		☐ No	
		☐ Doesn't know	
		☐ Refused to answer	

ID	Question	Answer(s)	Skip
Id10129	**Did (s)he have a recent negative test by a health professional for malaria?**		
Hint:	Remind the respondent that we are asking for the diagnosis assessed by a doctor, health worker, or other health professional during the final illness.		
		☐ Yes	
		☐ No	
		☐ Doesn't know	
		☐ Refused to answer	

ID	Question	Answer(s)	Skip
Id10130	**Was there any diagnosis by a health professional of dengue fever?**		
Hint:	Remind the respondent that we are asking for the diagnosis assessed by a doctor, health worker, or other health professional during the final illness.		
		☐ Yes	
		☐ No	
		☐ Doesn't know	
		☐ Refused to answer	

ID	Question	Answer(s)	Skip
Id10131	**Was there any diagnosis by a health professional of measles?**		
Hint:	Remind the respondent that we are asking for the diagnosis assessed by a doctor, health worker, or other health professional during the final illness.		
		☐ Yes	
		☐ No	
		☐ Doesn't know	
		☐ Refused to answer	

ID	Question	Answer(s)	Skip
Id10133	**Was there any diagnosis by a health professional of heart disease?**		
Hint:	Remind the respondent that we are asking for the diagnosis assessed by a doctor, health worker, or other health professional during the final illness.		
		☐ Yes	
		☐ No	
		☐ Doesn't know	
		☐ Refused to answer	

ID	Question	Answer(s)	Skip
Id10134	**Was there any diagnosis by a health professional of diabetes?**		
Hint:	Remind the respondent that we are asking for the diagnosis assessed by a doctor, health worker, or other health professional during the final illness.		
		☐ Yes	
		☐ No	
		☐ Doesn't know	
		☐ Refused to answer	

ID	Question	Answer(s)	Skip
Id10135	**Was there any diagnosis by a health professional of asthma?**		
Hint:	Remind the respondent that we are asking for the diagnosis assessed by a doctor, health worker, or other health professional during the final illness.		
		Yes	
		No	
		Doesn't know	
		Refused to answer	

ID	Question	Answer(s)	Skip
Id10136	**Was there any diagnosis by a health professional of epilepsy?**		
Hint:	Remind the respondent that we are asking for the diagnosis assessed by a doctor, health worker, or other health professional during the final illness.		
		Yes	
		No	
		Doesn't know	
		Refused to answer	

ID	Question	Answer(s)	Skip
Id10137	**Was there any diagnosis by a health professional of cancer?**		
Hint:	Remind the respondent that we are asking for the diagnosis assessed by a doctor, health worker, or other health professional during the final illness.		
		Yes	
		No	
		Doesn't know	
		Refused to answer	

ID	Question	Answer(s)	Skip
Id10142	**Was there any diagnosis by a health professional of sickle cell disease?**		
Hint:	Remind the respondent that we are asking for the diagnosis assessed by a doctor, health worker, or other health professional during the final illness.		
		Yes	
		No	
		Doesn't know	
		Refused to answer	

ID	Question	Answer(s)	Skip
Id10143	**Was there any diagnosis by a health professional of kidney disease?**		
Hint:	Remind the respondent that we are asking for the diagnosis assessed by a doctor, health worker, or other health professional during the final illness.		
		Yes	
		No	
		Doesn't know	
		Refused to answer	

ID	Question	Answer(s)	Skip
Id10144	Was there any diagnosis by a health professional of liver disease?		
Hint:	Remind the respondent that we are asking for the diagnosis assessed by a doctor, health worker, or other health professional during the final illness.		
		Yes	
		No	
		Doesn't know	
		Refused to answer	

ID	Question	Answer(s)	Skip
Id10147	Did (s)he have a fever?		
Hint:			
		Yes	
		No	10152
		Doesn't know	10152
		Refused to answer	10152

ID	Question	Answer(s)	Skip
Id10148	How many days did the fever last?		
Hint:	Less than 1 day = "0". Use 1 week = 7 days to determine the number of days.		
		Days	

ID	Question	Answer(s)	Skip
Id10149	Did the fever continue until death?		
Hint:			
		Yes	
		No	
		Doesn't know	
		Refused to answer	

ID	Question	Answer(s)	Skip
Id10150	How severe was the fever?		
Hint:			
		Mild	
		Moderate	
		Severe	

ID	Question	Answer(s)	Skip
Id10151	What was the pattern of the fever?		
Hint:			
		Continuous	
		On and off	
		Only at night	
		Doesn't know	
		Refused to answer	

ID	Question	Answer(s)	Skip
Id10152	Did (s)he have night sweats?		
Hint:			
		Yes	
		No	
		Doesn't know	
		Refused to answer	

ID	Question	Answer(s)	Skip
Id10153	Did (s)he have a cough?		
Hint:			
		Yes	
		No	10159
		Doesn't know	10159
		Refused to answer	10159

ID	Question	Answer(s)	Skip
Id10154	For how many days did (s)he have a cough?		
Hint:	Less than 1 day = “0”. Use 1 week = 7 days to determine the number of days.		
		Days	

ID	Question	Answer(s)	Skip
Id10155	Was the cough productive, with sputum?		
Hint:			
		Yes	
		No	
		Doesn't know	
		Refused to answer	

ID	Question	Answer(s)	Skip
Id10156	Was the cough very severe?		
Hint:			
		Yes	
		No	
		Doesn't know	
		Refused to answer	

ID	Question	Answer(s)	Skip
Id10157	Did (s)he cough up blood?		
Hint:			
		Yes	
		No	
		Doesn't know	
		Refused to answer	

ID	Question	Answer(s)	Skip
Id10158	Did (s)he make a whooping sound when coughing?		
Hint:			
		Yes	
		No	
		Doesn't know	
		Refused to answer	

ID	Question	Answer(s)	Skip
Id10159	Did (s)he have any difficulty breathing?		
Hint:			
		Yes	
		No	10166
		Doesn't know	10166
		Refused to answer	10166

ID	Question	Answer(s)	Skip
id10161_unit	How long did the difficulty breathing last?		
Hint:	Enter for one option only.		
		Days	
		Months	
		Years	

ID	Question	Answer(s)	Skip
Id10165	Was the difficulty continuous or on and off?		
Hint:			
		Continuous	
		On and off	
		Doesn't know	
		Refused to answer	

ID	Question	Answer(s)	Skip
Id10166	During the illness that led to death, did (s)he have fast breathing?		
Hint:			
		Yes	
		No	10168
		Doesn't know	10168
		Refused to answer	10168

ID	Question	Answer(s)	Skip
Id10167	For how many days did the fast breathing last?		
Hint:	Less than 1 day = “0”. Use 1 week = 7 days to determine the number of days.		
		Days	

ID	Question	Answer(s)	Skip
Id10168	Did (s)he have breathlessness?		
Hint:			
		Yes	
		No	10172
		Doesn't know	10172
		Refused to answer	10172

ID	Question	Answer(s)	Skip
Id10169	For how many days did (s)he have breathlessness?		
Hint:	Less than 1 day = “0.” Use 1 week = 7 days to determine the number of days.		
		Days	

ID	Question	Answer(s)	Skip
Id10172	Did you see the lower chest wall/ribs being pulled in as the child breathed in?		
Hint:	Ask for children under the age of 12 years. Show photos/video (if available).		
		Yes	
		No	
		Doesn't know	
		Refused to answer	

ID	Question	Answer(s)	Skip
Id10173	During the illness that led to death did his/her breathing sound like any of the following:		
Hint:	In case of adults demonstrate wheezing sound only because stridor and grunting is extremely rare in adults. Ask about wheezing, stridor and grunting only for children under the age of 12 years. Demonstrate each sound, play audio (if available), if in doubt select multiple.		
		Stridor	
		Grunting	
		Wheezing	
		No	
		Doesn't know	
		Refused to answer	

ID	Question	Answer(s)	Skip
Id10174	Did (s)he have chest pain?		
Hint:			
		Yes	
		No	10181
		Doesn't know	10181
		Refused to answer	10181

ID	Question	Answer(s)	Skip
Id10176	How many days before death did (s)he have chest pain?		
Hint:	Less than 1 day = “0.” Use 1 week = 7 days to determine the number of days.		
		Days	

ID	Question	Answer(s)	Skip
Id10181	**Did (s)he have more frequent loose or liquid stools than usual?**		
Hint:	Ask the respondent about his/her understanding of what is diarrhoea (having more frequent loose or liquid stools than usual); if unclear or wrong, explain to the respondent what is diarrhoea.		
		Yes	
		No	10186
		Doesn't know	10186
		Refused to answer	10186

ID	Question	Answer(s)	Skip
Id10182	**For how many days did (s)he have frequent loose or liquid stools?**		
Hint:	Less than 1 day= “0”. Use 1 week = 7 days to determine the number of days.		
		Days	

ID	Question	Answer(s)	Skip
Id10183	**How many stools did the baby or child have on the day that loose liquid stools were most frequent?**		
Hint:	Enter “99“ if this information is not known.		
		Number of stools:	

ID	Question	Answer(s)	Skip
Id10184	**How many days before death did the frequent loose or liquid stools start?**		
Hint:	Less than 1 day= “0”. Use 1 week = 7 days to determine the number of days. Enter “99“ if this information is not known.		
		Days	

ID	Question	Answer(s)	Skip
Id10185	**Did the frequent loose or liquid stools continue until death?**		
Hint:			
		Yes	
		No	
		Doesn't know	
		Refused to answer	

ID	Question	Answer(s)	Skip
Id10186	**At any time during the final illness was there blood in the stools?**		
Hint:			
		Yes	
		No	10188
		Doesn't know	10188
		Refused to answer	10188

ID	Question	Answer(s)	Skip
Id10187	**Was there blood in the stool up until death?**		
Hint:			
		Yes	
		No	
		Doesn't know	
		Refused to answer	

ID	Question	Answer(s)	Skip
Id10188	Did (s)he vomit?		
Hint:			
		Yes	
		No	
		Doesn't know	
		Refused to answer	

ID	Question	Answer(s)	Skip
Id10189	Did (s)he vomit in the week preceding the death?		
Hint:			
		Yes	
		No	10193
		Doesn't know	10193
		Refused to answer	10193

ID	Question	Answer(s)	Skip
Id10191	Was there blood in the vomit?		
Hint:			
		Yes	
		No	
		Doesn't know	
		Refused to answer	

ID	Question	Answer(s)	Skip
Id10192	Was the vomit black?		
Hint:			
		Yes	
		No	
		Doesn't know	
		Refused to answer	

ID	Question	Answer(s)	Skip
Id10193	Did (s)he have any belly (abdominal) problem?		
Hint:	Explain to the respondent that problems could be pain, protruding abdomen or a mass.		
		Yes	
		No	
		Doesn't know	
		Refused to answer	

ID	Question	Answer(s)	Skip
Id10194	Did (s)he have belly (abdominal) pain?		
Hint:			
		Yes	
		No	10200
		Doesn't know	10200
		Refused to answer	10200

ID	Question	Answer(s)	Skip
Id10195	Was the belly (abdominal) pain severe?		
Hint:			
		Yes	
		No	10200
		Doesn't know	10200
		Refused to answer	10200

ID	Question	Answer(s)	Skip
pain_duration	For how long did (s)he have belly (abdominal) pain?		
Hint:	Enter for one option only.		
		Hours	
		Days	
		Weeks	
		Months	

ID	Question	Answer(s)	Skip
Id10199	Was the pain in the upper or lower belly (abdomen)?		
Hint:			
		Upper abdomen	
		Lower abdomen	
		Upper and lower abdomen	
		Doesn't know	
		Refused to answer	

ID	Question	Answer(s)	Skip
Id10200	Did (s)he have a more than usually protruding belly (abdomen)?		
Hint:			
		Yes	
		No	10204
		Doesn't know	10204
		Refused to answer	10204

ID	Question	Answer(s)	Skip
Id10201	For how many days did (s)he have a more than usually protruding belly (abdomen)?		
Hint:	Less than 1 day= “0”. Use 1 week = 7 days to determine the number of days.		
		Days	

ID	Question	Answer(s)	Skip
Id10202	For how many months did (s)he have a more than usually protruding belly (abdomen)?		
Hint:			
		Months	

ID	Question	Answer(s)	Skip
Id10203	How rapidly did (s)he develop the protruding belly (abdomen)?		
Hint:			
		Rapidly	
		Slowly	

ID	Question	Answer(s)	Skip
Id10204	Did (s)he have any mass in the belly (abdomen)?		
Hint:			
		Yes	
		No	10207
		Doesn't know	10207
		Refused to answer	10207

ID	Question	Answer(s)	Skip
Id10205	For how many days before death did (s)he have a mass in the belly (abdomen)?		
Hint:	Less than 1 day= “0”. Use 1 week = 7 days to determine the number of days.		
		Days	

ID	Question	Answer(s)	Skip
Id10206	For how many months before death did (s)he have a mass in the belly (abdomen)?		
Hint:	Less than 1 month, report in days. 1 month=30 days		
		Months	

ID	Question	Answer(s)	Skip
Id10207	Did (s)he have a severe headache?		
Hint:			
		Yes	
		No	
		Doesn't know	
		Refused to answer	

ID	Question	Answer(s)	Skip
Id10208	Did (s)he have a stiff neck during illness that led to death?		
Hint:			
		Yes	
		No	10210
		Doesn't know	10210
		Refused to answer	10210

ID	Question	Answer(s)	Skip
Id10209	For how many days before death did (s)he have stiff neck?		
Hint:	Less than 1 day= “0”. 1 week= 7 days. 1 month=30 days		
		Days	

ID	Question	Answer(s)	Skip
Id10210	Did (s)he have a painful neck during the illness that led to death?		
Hint:			
		Yes	
		No	10214
		Doesn't know	10214
		Refused to answer	10214

ID	Question	Answer(s)	Skip
Id10211	For how many days before death did (s)he have a painful neck?		
Hint:	Less than 1 day= “0”. 1 week= 7 days. 1 month=30 days		
		Days	

ID	Question	Answer(s)	Skip
Id10214	Was (s)he unconscious during the illness that lead to death?		
Hint:			
		Yes	
		No	10219
		Doesn't know	10219
		Refused to answer	10219

ID	Question	Answer(s)	Skip
Id10215	Was (s)he unconscious for more than 24 hours before death?		
Hint:			
		Yes	10217
		No	
		Doesn't know	
		Refused to answer	

ID	Question	Answer(s)	Skip
Id10216	How many hours before death did unconsciousness start?		
Hint:	The question needs input in hours but the respondent may not know exactly and so it may be easier to ask 'how long' and then convert the duration in hours. (Less than 1 hour = "0")		
		Hours	

ID	Question	Answer(s)	Skip
Id10217	Did the unconsciousness start suddenly, quickly (at least within a single day)?		
Hint:			
		Yes	
		No	
		Doesn't know	
		Refused to answer	

ID	Question	Answer(s)	Skip
Id10218	Did the unconsciousness continue until death?		
Hint:			
		Yes	
		No	
		Doesn't know	
		Refused to answer	

ID	Question	Answer(s)	Skip
Id10219	Did (s)he have convulsions?		
Hint:			
		Yes	
		No	10223
		Doesn't know	10223
		Refused to answer	10223

ID	Question	Answer(s)	Skip
Id10220	Did (s)he experience any generalized convulsions or fits during the illness that led to death?		
Hint:			
		Yes	
		No	
		Doesn't know	
		Refused to answer	

ID	Question	Answer(s)	Skip
Id10221	For how many minutes did the convulsions last?		
Hint:	Less than 1 minute = "0". Use 1 hour=60 minutes to determine the number of minutes		
		Minutes	

ID	Question	Answer(s)	Skip
Id10222	Did (s)he become unconscious immediately after the convulsions?		
Hint:			
		Yes	
		No	
		Doesn't know	
		Refused to answer	

ID	Question	Answer(s)	Skip
Id10223	Did (s)he have any urine problems?		
Hint:	Explain to the respondent that urine problems refer to urinating a lot or not at all, and blood in the urine.		
		Yes	
		No	10227
		Doesn't know	10227
		Refused to answer	10227

ID	Question	Answer(s)	Skip
Id10224	Did (s)he stop urinating?		
Hint:	This means that the deceased stopped urinating.		
		Yes	
		No	
		Doesn't know	
		Refused to answer	

ID	Question	Answer(s)	Skip
Id10225	Did (s)he go to urinate more often than usual?		
Hint:			
		Yes	
		No	
		Doesn't know	
		Refused to answer	

ID	Question	Answer(s)	Skip
Id10226	During the final illness did (s)he ever pass blood in the urine?		
Hint:			
		Yes	
		No	
		Doesn't know	
		Refused to answer	

ID	Question	Answer(s)	Skip
Id10227	Did (s)he have sores or ulcers anywhere on the body?		
Hint:			
		Yes	
		No	10230
		Doesn't know	10230
		Refused to answer	10230

ID	Question	Answer(s)	Skip
Id10229	Did the sores have clear fluid or pus?		
Hint:			
		Yes	
		No	
		Doesn't know	
		Refused to answer	

ID	Question	Answer(s)	Skip
Id10230	Did (s)he have an ulcer (pit) on the foot?		
Hint:			
		Yes	
		No	10233
		Doesn't know	10233
		Refused to answer	10233

ID	Question	Answer(s)	Skip
Id10231	Did the ulcer on the foot ooze pus?		
Hint:			
		Yes	
		No	10233
		Doesn't know	10233
		Refused to answer	10233

ID	Question	Answer(s)	Skip
Id10232	For how many days did the ulcer on the foot ooze pus?		
Hint:	Less than 1 day= “0”. Use 1 week=7 days to determine the number of days.		
		Days	

ID	Question	Answer(s)	Skip
Id10233	During the illness that led to death, did (s)he have any skin rash?		
Hint:			
		Yes	
		No	10238
		Doesn't know	10238
		Refused to answer	10238

ID	Question	Answer(s)	Skip
Id10234	For how many days did (s)he have the skin rash?		
Hint:	Less than 1 day= “0”. Use 1 week=7 days to determine the number of days		
		Days	

ID	Question	Answer(s)	Skip
Id10235	Where was the rash?		
Hint:	Select all that apply.		
		Face	
		Trunk or abdomen	
		Extremities	
		Everywhere	

ID	Question	Answer(s)	Skip
Id10236	Did (s)he have measles rash (use local term)?		
Hint:			
		Yes	
		No	
		Doesn't know	
		Refused to answer	

ID	Question	Answer(s)	Skip
Id10238	During the illness that led to death, did her/his skin flake off in patches?		
Hint:			
		Yes	
		No	
		Doesn't know	
		Refused to answer	

ID	Question	Answer(s)	Skip
Id10239	During the illness that led to death, did he/she have areas of the skin that turned black?		
Hint:		Yes	
		No	
		Doesn't know	
		Refused to answer	

ID	Question	Answer(s)	Skip
Id10240	During the illness that led to death, did he/she have areas of the skin with redness and swelling?		
Hint:		Yes	
		No	
		Doesn't know	
		Refused to answer	

ID	Question	Answer(s)	Skip
Id10241	During the illness that led to death, did (s)he bleed from anywhere?		
Hint:		Yes	
		No	10243
		Doesn't know	10243
		Refused to answer	10243

ID	Question	Answer(s)	Skip
Id10242	Did (s)he bleed from the nose, mouth or anus?		
Hint:		Yes	
		No	
		Doesn't know	
		Refused to answer	

ID	Question	Answer(s)	Skip
Id10243	Did (s)he have noticeable weight loss?		
Hint:		Yes	
		No	
		Doesn't know	
		Refused to answer	

ID	Question	Answer(s)	Skip
Id10244	Was (s)he severely thin or wasted?		
Hint:			
		Yes	
		No	
		Doesn't know	
		Refused to answer	

ID	Question	Answer(s)	Skip
Id10245	During the illness that led to death, did s/he have a whitish rash inside the mouth or on the tongue?		
Hint:			
		Yes	
		No	
		Doesn't know	
		Refused to answer	

ID	Question	Answer(s)	Skip
Id10246	Did (s)he have stiffness of the whole body or was unable to open the mouth?		
Hint:			
		Yes	
		No	
		Doesn't know	
		Refused to answer	

ID	Question	Answer(s)	Skip
Id10247	Did (s)he have puffiness of the face?		
Hint:			
		Yes	
		No	10249
		Doesn't know	10249
		Refused to answer	10249

ID	Question	Answer(s)	Skip
Id10248	For how many days did (s)he have puffiness of the face?		
Hint:	Less than 1 day= "0". Use 1 month= 7 days or 1 month=30 days to determine the number of days.		
		Days	

ID	Question	Answer(s)	Skip
Id10249	During the illness that led to death, did (s)he have swollen legs or feet?		
Hint:			
		Yes	
		No	10252
		Doesn't know	10252
		Refused to answer	10252

ID	Question	Answer(s)	Skip
Id10250	How many days did the swelling last?		
Hint:	Less than 1 day="0". Use 1 month=30 days or 1 month=30 days to determine the number of days.		
		Days	

ID	Question	Answer(s)	Skip
Id10251	Did (s)he have both feet swollen?		
Hint:		Yes	
		No	
		Doesn't know	
		Refused to answer	

ID	Question	Answer(s)	Skip
Id10252	Did (s)he have general puffiness all over his/her body?		
Hint:		Yes	
		No	
		Doesn't know	
		Refused to answer	

ID	Question	Answer(s)	Skip
Id10253	Did (s)he have any lumps?		
Hint:		Yes	
		No	10258
		Doesn't know	10258
		Refused to answer	10258

ID	Question	Answer(s)	Skip
Id10255	Did (s)he have any lumps on the neck?		
Hint:		Yes	
		No	
		Doesn't know	
		Refused to answer	

ID	Question	Answer(s)	Skip
Id10256	Did (s)he have any lumps on the armpit?		
Hint:		Yes	
		No	
		Doesn't know	
		Refused to answer	

ID	Question	Answer(s)	Skip
Id10257	Did (s)he have any lumps on the groin?		
Hint:			
		Yes	
		No	
		Doesn't know	
		Refused to answer	

ID	Question	Answer(s)	Skip
Id10258	Was (s)he in any way paralysed?		
Hint:			
		Yes	
		No	10261
		Doesn't know	10261
		Refused to answer	10261

ID	Question	Answer(s)	Skip
Id10259	Did s(he) have paralysis of only one side of the body?		
Hint:			
		Yes	
		No	
		Doesn't know	
		Refused to answer	

ID	Question	Answer(s)	Skip
Id10260	Which were the limbs or body parts paralysed?		
Hint:	Select all that apply.		
		Right side	
		Left side	
		Lower part of body	
		Upper part of body	
		One leg only	
		One arm only	
		Whole body	
		Other	

ID	Question	Answer(s)	Skip
Id10261	Did (s)he have difficulty swallowing?		
Hint:			
		Yes	
		No	10264
		Doesn't know	10264
		Refused to answer	10264

ID	Question	Answer(s)	Skip
Id10262	For how many days before death did (s)he have difficulty swallowing?		
Hint:	Less than 1 day="0". Use 1 month=30 days or 1 month=30 days to determine the number of days		
		Days	

ID	Question	Answer(s)	Skip
Id10263	Was the difficulty with swallowing with solids, liquids, or both?		
Hint:			
		Solids	
		Liquids	
		Both	

ID	Question	Answer(s)	Skip
Id10264	Did (s)he have pain upon swallowing?		
Hint:			
		Yes	
		No	
		Doesn't know	
		Refused to answer	

ID	Question	Answer(s)	Skip
Id10265	Did (s)he have yellow discoloration of the eyes?		
Hint:			
		Yes	
		No	10267
		Doesn't know	10267
		Refused to answer	10267

ID	Question	Answer(s)	Skip
Id10266	For how many days did (s)he have the yellow discoloration?		
Hint:	Less than 1 day="0". Use 1 month=30 days or 1 month=30 days to determine the number of days		
		Days	

ID	Question	Answer(s)	Skip
Id10267	Did her/his hair change in color to a reddish or yellowish color?		
Hint:			
		Yes	
		No	
		Doesn't know	
		Refused to answer	

ID	Question	Answer(s)	Skip
Id10268	Did (s)he look pale (thinning/lack of blood) or have pale palms, eyes or nail beds?		
Hint:	Long term deficiency of the blood results in a pale, whitish appearance of the lips, tongue, and eye sac. Sometime it is referred to as thinning or lack of blood, or pallor.		
		☐ Yes	
		☐ No	
		☐ Doesn't know	
		☐ Refused to answer	

ID	Question	Answer(s)	Skip
Id10269	Did (s)he have sunken eyes?		
Hint:			
		☐ Yes	
		☐ No	
		☐ Doesn't know	
		☐ Refused to answer	

ID	Question	Answer(s)	Skip
Id10270	Did (s)he drink a lot more water than usual?		
Hint:			
		☐ Yes	
		☐ No	
		☐ Doesn't know	
		☐ Refused to answer	

ID	Question	Answer(s)	Skip
Id10271	Was the baby able to suckle or bottle-feed within the first 24 hours after birth?		
Hint:	Ask only if the child was < 1 year old when it died.		
		☐ Yes	
		☐ No	
		☐ Doesn't know	
		☐ Refused to answer	

ID	Question	Answer(s)	Skip
Id10272	Did the baby ever suckle in a normal way?		
Hint:	Ask only if the child was < 1 year old when it died.		
		☐ Yes	
		☐ No	
		☐ Doesn't know	
		☐ Refused to answer	

ID	Question	Answer(s)	Skip
Id10273	Did the baby stop suckling?		
Hint:	Ask only if the child was < 1 year old when it died.		
		Yes	
		No	10275
		Doesn't know	10275
		Refused to answer	10275

ID	Question	Answer(s)	Skip
Id10274	How many days after birth did the baby stop suckling?		
Hint:	Ask only if the child was < 1 year old when it died.		
		Days	

ID	Question	Answer(s)	Skip
Id10275	Did the baby have convulsions starting within the first 24 hours of life?		
Hint:	Ask only if the child was < 1 year old when it died.		
		Yes	10277
		No	
		Doesn't know	
		Refused to answer	

ID	Question	Answer(s)	Skip
Id10276	Did the baby have convulsions starting more than 24 hours after birth?		
Hint:	Ask only if the child was < 1 year old when it died.		
		Yes	
		No	
		Doesn't know	
		Refused to answer	

ID	Question	Answer(s)	Skip
Id10277	Did the baby's body become stiff, with the back arched backwards?		
Hint:	Ask only if the child was < 1 year old when it died.		
		Yes	
		No	
		Doesn't know	
		Refused to answer	

ID	Question	Answer(s)	Skip
Id10278	During the illness that led to death, did the baby have a bulging or raised fontanelle? (ask only up to 18 months)		
Hint:	Show photo (if available)		
		Yes	10281
		No	
		Doesn't know	
		Refused to answer	

ID	Question	Answer(s)	Skip
Id10279	During the illness that led to death, did the baby have a sunken fontanelle? (ask only up to 18 months)		
Hint:	Show photo (if available)		
		Yes	
		No	
		Doesn't know	
		Refused to answer	

ID	Question	Answer(s)	Skip
Id10281	During the illness that led to death, did the baby become unresponsive or unconscious?		
Hint:			
		Yes	
		No	
		Doesn't know	
		Refused to answer	

ID	Question	Answer(s)	Skip
Id10282	Did the baby become unresponsive or unconscious soon after birth, within less than 24 hours?		
Hint:			
		Yes	
		No	
		Doesn't know	
		Refused to answer	

ID	Question	Answer(s)	Skip
Id10283	Did the baby become unresponsive or unconscious more than 24 hours after birth?		
Hint:			
		Yes	
		No	
		Doesn't know	
		Refused to answer	

ID	Question	Answer(s)	Skip
Id10352	How many years old was the child when the fatal illness started?		
Hint:	If child < 1 year, fill in "0."		
		Years	

ID	Question	Answer(s)	Skip
Id10354	Was the child part of a multiple birth?		
Hint:	If two or more children are born at the same time, it is counted as a multiple birth, even if one or more of the babies are born dead.		
		Yes	
		No	10356
		Doesn't know	10356
		Refused to answer	10356

ID	Question	Answer(s)	Skip
Id10355	Was the child the first, second, or later in the birth order?		
Hint:			
		First	
		Second or later	

ID	Question	Answer(s)	Skip
Id10356	Is the mother still alive?		
Hint:	If mother is present at the interview, select 'yes' without asking the question aloud. Only read this question if the respondent is not the mother and if it is not yet known if the mother is alive.		
		Yes	10360
		No	
		Doesn't know	
		Refused to answer	

ID	Question	Answer(s)	Skip
Id10357	Did the mother die during or after the delivery?		
Hint:			
		During delivery	10360
		After delivery	

ID	Question	Answer(s)	Skip
mother_death	How long after the delivery did the mother die?		
Hint:	The respondent may reply in months or days. If less than 24 hours, record "0" days. If < 1 month, record in days; if less than < 2 months, record in weeks; if ≥ 2 months, record in completed months.		
		Months	
		Weeks	
		Days	

ID	Question	Answer(s)	Skip
Id10360	Where was the deceased born?		
Hint:	Read the question and slowly read the first 5 choices. Respondent should hear all 5 choices and then respond.		
		Hospital	
		Other health facility	
		Home	
		On route to hospital or facility	
		Other	
		Doesn't know	
		Refused to answer	

ID	Question	Answer(s)	Skip
Id10361	**Did you/the mother receive professional assistance during the delivery? (ask only up to 1 year)**		
Hint:	Explain to the respondent what is meant by professional assistance: delivery attended by a medical professional (doctor, nurse or midwife).		
		Yes	
		No	
		Doesn't know	
		Refused to answer	

ID	Question	Answer(s)	Skip
Id10362	**At birth, was the baby of usual size?**		
Hint:	Show photos (if available), explain to the respondent that even if the answer is "no" some more questions will be asked, just to make sure no important detail has been missed.		
		Yes	10366
		No	
		Doesn't know	
		Refused to answer	

ID	Question	Answer(s)	Skip
Id10363	**At birth, was the baby smaller than usual, (weighing under 2.5 kg)?**		
Hint:	Show photos (if available).		
		Yes	
		No	10365
		Doesn't know	10365
		Refused to answer	10365

ID	Question	Answer(s)	Skip
Id10364	**At birth, was the baby very much smaller than usual, (weighing under 1 kg)?**		
Hint:	Show photos (if available).		
		Yes	10366
		No	10366
		Doesn't know	10366
		Refused to answer	10366

ID	Question	Answer(s)	Skip
Id10365	**At birth, was the baby larger than usual, (weighing over 4.5 kg)?**		
Hint:	Show photos (if available).		
		Yes	
		No	
		Doesn't know	
		Refused to answer	

ID	Question	Answer(s)	Skip
Id10366	**What was the weight (in grammes) of the deceased at birth?**		
Hint:	Respondents may give the answer in kilograms. For the data entry, convert to grammes. 1 kilogram=1,000 grammes. Enter "9999" for "Don't know." Enter "8888" for "Refused to answer."		
		Grammes:	

ID	Question	Answer(s)	Skip
Id10367	**How many months long was the pregnancy before the child was born? (ask only up to 1 year)**		
Hint:	If don't know enter "99"		
		Months	

ID	Question	Answer(s)	Skip
Id10368	**Were there any complications in the late part of the pregnancy (defined as the last 3 months, before labour)? (ask only up to 1 year)**		
Hint:			
		Yes	
		No	
		Doesn't know	
		Refused to answer	

ID	Question	Answer(s)	Skip
Id10369	**Were there any complications during labour or delivery? (ask only up to 1 year)**		
Hint:			
		Yes	
		No	
		Doesn't know	
		Refused to answer	

ID	Question	Answer(s)	Skip
Id10370	**Was any part of the baby physically abnormal at time of delivery? (for example: body part too large or too small, additional growth on body)?**		
Hint:			
		Yes	
		No	10418
		Doesn't know	
		Refused to answer	

ID	Question	Answer(s)	Skip
Id10371	**Did the baby/ child have a swelling or defect on the back at time of birth?**		
Hint:			
		Yes	
		No	
		Doesn't know	
		Refused to answer	

ID	Question	Answer(s)	Skip
Id10372	Did the baby/ child have a very large head at time of birth?		
Hint:			
		Yes	
		No	
		Doesn't know	
		Refused to answer	

ID	Question	Answer(s)	Skip
Id10373	Did the baby/ child have a very small head at time of birth?		
Hint:			
		Yes	
		No	
		Doesn't know	
		Refused to answer	

ID	Question	Answer(s)	Skip
Id10408_1	Before the illness that led to death, was the baby/the child growing normally?		
Hint:			
		Yes	
		No	
		Doesn't know	
		Refused to answer	

ID	Question	Answer(s)	Skip
Id10418	Did (s)he receive any treatment for the illness that led to death?		
Hint:			
		Yes	
		No	10428
		Doesn't know	10428
		Refused to answer	10428

ID	Question	Answer(s)	Skip
Id10419	Did (s)he receive oral rehydration salts?		
Hint:			
		Yes	
		No	
		Doesn't know	
		Refused to answer	

ID	Question	Answer(s)	Skip
Id10420	Did (s)he receive (or need) intravenous fluids (drip) treatment?		
Hint:			
		Yes	
		No	
		Doesn't know	
		Refused to answer	

ID	Question	Answer(s)	Skip
Id10421	Did (s)he receive (or need) a blood transfusion?		
Hint:			
		Yes	
		No	
		Doesn't know	
		Refused to answer	

ID	Question	Answer(s)	Skip
Id10422	Did (s)he receive (or need) treatment/food through a tube passed through the nose?		
Hint:			
		Yes	
		No	
		Doesn't know	
		Refused to answer	

ID	Question	Answer(s)	Skip
Id10423	Did (s)he receive (or need) injectable antibiotics?		
Hint:			
		Yes	
		No	
		Doesn't know	
		Refused to answer	

ID	Question	Answer(s)	Skip
Id10424	Did (s)he receive (or need) antiretroviral therapy (ART)?		
Hint:			
		Yes	
		No	
		Doesn't know	
		Refused to answer	

ID	Question	Answer(s)	Skip
Id10425	Did (s)he have (or need) an operation for the illness?		
Hint:			
		Yes	
		No	10427
		Doesn't know	10427
		Refused to answer	10427

ID	Question	Answer(s)	Skip
Id10426	Did (s)he have the operation within 1 month before death?		
Hint:			
		Yes	
		No	
		Doesn't know	
		Refused to answer	

ID	Question	Answer(s)	Skip
Id10427	Was (s)he discharged from hospital very ill?		
Hint:			
		Yes	
		No	
		Doesn't know	
		Refused to answer	

ID	Question	Answer(s)	Skip
Id10428	Had (s)he received immunizations?		
Hint:			
		Yes	
		No	10432
		Doesn't know	10432
		Refused to answer	10432

ID	Question	Answer(s)	Skip
Id10429	Do you have the child's vaccination card?		
Hint:			
		Yes	
		No	10432
		Doesn't know	10432
		Refused to answer	10432

ID	Question	Answer(s)	Skip
Id10430	Can I see the vaccination card (note the vaccines the child received)?		
Hint:	Only fill in the vaccination sheet if the respondent has the vaccination card at the present moment during the VA interview; the interviewer should fill in the vaccination sheet, not the respondent. If applicable, take photo with name covered.		
		Yes	
		No	10432
		Doesn't know	10432
		Refused to answer	10432

ID	Question	Answer(s)	Skip
Id10431	[Note vaccines here]		
Hint:	If applicable, record vaccine, date, and age of child		

ID	Question	Answer(s)	Skip
Id10432	Was care sought outside the home while (s)he had this illness?		
Hint:		☐ Yes	
		☐ No	10450
		☐ Doesn't know	10450
		☐ Refused to answer	10450

ID	Question	Answer(s)	Skip
Id10433	**Where or from whom did you seek care?**		
Hint:	Read the question and read the choices. Select all that apply.		
		☐ Traditional healer	
		☐ Homeopath	
		☐ Religious leader	
		☐ Government hospital	
		☐ Government health center or clinic	
		☐ Private hospital	
		☐ Community-based practitioner associated with health system	
		☐ Trained birth attendant	
		☐ Private physician	
		☐ Relative, friend (outside household)	
		☐ Pharmacy	
		☐ Doesn't know	
		☐ Refused to answer	

ID	Question	Answer(s)	Skip
Id10434	**Record the name and address of any hospital, health center or clinic where care was sought**		
Hint:	Record information provided.		

ID	Question	Answer(s)	Skip
Id10435	**Did a health care worker tell you the cause of death?**		
Hint:			
		☐ Yes	
		☐ No	10437
		☐ Doesn't know	10437
		☐ Refused to answer	10437

ID	Question	Answer(s)	Skip
Id10436	What did the health care worker say?		
Hint:			

ID	Question	Answer(s)	Skip
Id10437	Do you have any health records that belonged to the deceased?		
Hint:			
		Yes	
		No	10445
		Doesn't know	10445
		Refused to answer	10445

ID	Question	Answer(s)	Skip
Id10438	Can I see the health records?		
Hint:			
		Yes	
		No	10445
		Doesn't know	10445
		Refused to answer	10445

ID	Question	Answer(s)	Skip
Id10439	[Record the date of the most recent (last) visit]		
Hint:	If date unknown, record January 1, 2000 to indicate not available.		
		Day/ Month/ Year/	

ID	Question	Answer(s)	Skip
Id10445	Have you/has the deceased's (biological) mother ever been tested for HIV?		
Hint:			
		Yes	
		No	
		Doesn't know	
		Refused to answer	

ID	Question	Answer(s)	Skip
Id10446	Have you/has the deceased's (biological) mother ever been told she had HIV/AIDS by a health worker?		
Hint:			
		Yes	
		No	
		Doesn't know	
		Refused to answer	

ID	Question	Answer(s)	Skip
Id10450	In the final days before death, did s/he travel to a hospital or health facility?		
Hint:			
		Yes	
		No	10455
		Doesn't know	10455
		Refused to answer	10455

ID	Question	Answer(s)	Skip
Id10451	Did (s)he use motorised transport to get to the hospital or health facility?		
Hint:			
		Yes	
		No	
		Doesn't know	
		Refused to answer	

ID	Question	Answer(s)	Skip
Id10452	Were there any problems during admission to the hospital or health facility?		
Hint:			
		Yes	
		No	
		Doesn't know	
		Refused to answer	

ID	Question	Answer(s)	Skip
Id10453	Were there any problems with the way (s)he was treated (medical treatment, procedures, interpersonal attitudes, respect, dignity) in the hospital or health facility?		
Hint:			
		Yes	
		No	
		Doesn't know	
		Refused to answer	

ID	Question	Answer(s)	Skip
Id10454	Were there any problems getting medications or diagnostic tests in the hospital or health facility?		
Hint:			
		Yes	
		No	
		Doesn't know	
		Refused to answer	

ID	Question	Answer(s)	Skip
Id10455	Does it take more than 2 hours to get to the nearest hospital or health facility from the deceased's household?		
Hint:			
		Yes	
		No	
		Doesn't know	
		Refused to answer	

ID	Question	Answer(s)	Skip
Id10456	In the final days before death, were there any doubts about whether medical care was needed?		
Hint:			
		Yes	
		No	
		Doesn't know	
		Refused to answer	

ID	Question	Answer(s)	Skip
Id10457	In the final days before death, was traditional medicine used?		
Hint:			
		Yes	
		No	
		Doesn't know	
		Refused to answer	

ID	Question	Answer(s)	Skip
Id10458	In the final days before death, did anyone use a telephone or cell phone to call for help?		
Hint:			
		Yes	
		No	
		Doesn't know	
		Refused to answer	

ID	Question	Answer(s)	Skip
Id10459	Over the course of illness, did the total costs of care and treatment prohibit other household payments?		
Hint:			
		Yes	
		No	
		Doesn't know	
		Refused to answer	

ID	Question	Answer(s)	Skip
Id10462	Was a death certificate issued?		
Hint:	The following information serves only to complete cause of death information in some environments. In routine CRVS this information could be skipped in the interview, and information be collected from other sources, if available.		
		Yes	
		No	10476
		Doesn't know	10476
		Refused to answer	10476

ID	Question	Answer(s)	Skip
Id10463	Can I see the death certificate?		
Hint:	Fill in the following questions only if you are shown the copy of the certificate. Do not fill in just based on oral statements.		
		Yes	
		No	10476
		Doesn't know	10476
		Refused to answer	10476

ID	Question	Answer(s)	Skip
Id10464	[Record the immediate cause of death from the certificate (line 1a)]		
Hint:	An antecedent cause is the one that caused the one on the line above, e.g. diabetes mellitus may be an antecedent cause to kidney disease.		

ID	Question	Answer(s)	Skip
Id10465	[Duration (Ia):]		
Hint:			

ID	Question	Answer(s)	Skip
Id10466	[Record the first antecedent cause of death from the certificate (line 1b)]		
Hint:	For all following lines, add duration, if stated.		

ID	Question	Answer(s)	Skip
Id10467	[Duration (Ib):]		
Hint:			

ID	Question	Answer(s)	Skip
Id10468	[Record the second antecedent cause of death from the certificate (line 1c)]		
Hint:			

ID	Question	Answer(s)	Skip
Id10469	[Duration (Ic):]		
Hint:			

ID	Question	Answer(s)	Skip
Id10470	[Record the third antecedent cause of death from the certificate (line 1d)]		
Hint:			

ID	Question	Answer(s)	Skip
Id10471	[Duration (Id):]		
Hint:			

ID	Question	Answer(s)	Skip
Id10472	[Record the contributing cause(s) of death from the certificate (part 2)]		
Hint:			

ID	Question	Answer(s)	Skip
Id10473	[Duration (part2):]		
Hint:			

ID	Question	Answer(s)	Skip
Id10476	**NARRATIVE DESCRIPTION Thank you for your information. Now can you please tell me in your own words about the events that led to the death?**		
Hint:	AUDIO RECORDING • IF APPLICABLE, REQUEST CONSENT FOR AUDIO RECORDING: May I record your response? • IF YES, RECORD VERBAL CONSENT AT START OF RECORDING: "Do I have consent to record your response? WRITTEN NOTES AND PROBING • RECORD DETAILED NOTES OF RESPONSE; USE ADDITIONAL PAPER AS NEEDED. • IF NEEDED, PROBE FOR ADDL DETAILS ON WHEN RESPONDENT RECOGNIZED SYMPTOMS, CARE SOUGHT, BARRIERS TO CARE, ISSUES WITH TRANSPORT, ABNORMALITIES, ETC.		

ID	Question	Answer(s)	Skip
Id10478	Select any of the following words that were mentioned as present in the narrative.		
Hint:			
		☐ Abdomen	
		☐ Cancer	
		☐ Dehydration	
		☐ Dengue fever	
		☐ Diarrhoea	
		☐ Fever	
		☐ Heart problems	
		☐ Jaundice (yellow skin or eyes)	
		☐ Pneumonia	
		☐ Rash	
		☐ None of the above words were mentioned	
		☐ Don't know	

ID	Question	Answer(s)	Skip
Id10481	Time at end of interview		
Hint:	hh:mm 24 h		
		HH: ☐☐ MM: ☐☐	

ID	Question	Answer(s)	Skip
form_end	End of the Interview - Thanks		
Hint:			
